# Liquid-infused microstructured bioadhesives halt non-compressible hemorrhage

**DOI:** 10.1038/s41467-022-32803-1

**Published:** 2022-08-26

**Authors:** Guangyu Bao, Qiman Gao, Massimo Cau, Nabil Ali-Mohamad, Mitchell Strong, Shuaibing Jiang, Zhen Yang, Amin Valiei, Zhenwei Ma, Marco Amabili, Zu-Hua Gao, Luc Mongeau, Christian Kastrup, Jianyu Li

**Affiliations:** 1 Department of Mechanical Engineering, McGill University, Montreal, QC Canada; 2 Faculty of Dentistry, McGill University, Montreal, QC Canada; 3 Michael Smith Laboratories, University of British Columbia, Vancouver, BC Canada; 4 Department of Chemical Engineering, McGill University, Montreal, QC Canada; 5 Department of Pathology & Laboratory Medicine, University of British Columbia, Vancouver, BC Canada; 6 Blood Research Institute, Versiti, Milwaukee, WI USA; 7 Department of Surgery, Division of Trauma and Acute Care Surgery, Medical College of Wisconsin, Milwaukee, WI USA; 8 Department of Biochemistry, Medical College of Wisconsin, Milwaukee, WI USA; 9 Department of Biomedical Engineering, Medical College of Wisconsin, Milwaukee, WI USA; 10 Department of Pharmacology and Toxicology, Medical College of Wisconsin, Milwaukee, WI USA; 11 Department of Biomedical Engineering, McGill University, Montreal, QC Canada; 12 Department of Surgery, McGill University, Montreal, QC Canada

**Keywords:** Biomedical engineering, Bioinspired materials, Mechanical engineering, Polymers, Bioinspired materials

## Abstract

Non-compressible hemorrhage is an unmet clinical challenge that accounts for high mortality in trauma. Rapid pressurized blood flows under hemorrhage impair the function and integrity of hemostatic agents and the adhesion of bioadhesive sealants. Here, we report the design and performance of bioinspired microstructured bioadhesives, formed with a macroporous tough xerogel infused with functional liquids. The xerogel can rapidly absorb interfacial fluids such as whole blood and promote blood clotting, while the infused liquids facilitate interfacial bonding, sealing, and antibacterial function. Their synergy enables the bioadhesives to form tough adhesion on ex vivo human and porcine tissues and diverse engineered surfaces without the need for compression, as well as on-demand instant removal and storage stability. We demonstrate a significantly improved hemostatic efficacy and biocompatibility in rats and pigs compared to non-structured counterparts and commercial products. This work opens new avenues for the development of bioadhesives and hemostatic sealants.

## Abstract (web summary)

Non‐compressible wounds are a major source of high mortality in trauma victims. Here the authors report on the creation of xerogels impregnated with liquid adhesives which can rapidly absorb fluids promoting blood clotting while forming adhesions to tissue and demonstrate the xerogel in ex vivo and in vivo models.

## Introduction

Uncontrolled hemorrhage accounts for more than 30% of trauma deaths^1,2^. Despite tremendous research efforts, critical challenges remain for treating non-compressible and deep-narrow hemorrhages, which present rapid pressurized blood flows from wound sites^3,4^. Common strategies reliant on hemostatic agents alone, such as thrombin and kaolin, to promote blood clotting are limited by slow clotting rate and coagulopathies^5^. Alternative strategies are bioadhesive sealants that block the bleeding site physically^6–10^. Removal of interfacial fluids is critical for adhesion formation and sealing performance of the bioadhesives^11^. However, existing bioadhesives are slow and ineffective in removing the rapid pressurized blood at the interface due to their non- and nano-porous structures^12,13^. Situations in point-of-care and emergency rooms impose other requirements such as easy-to-use and stability of storage that are often overlooked^1^. Addressing these challenges calls for new designs and materials for non-compressible hemorrhage.

**Fig. 1:**
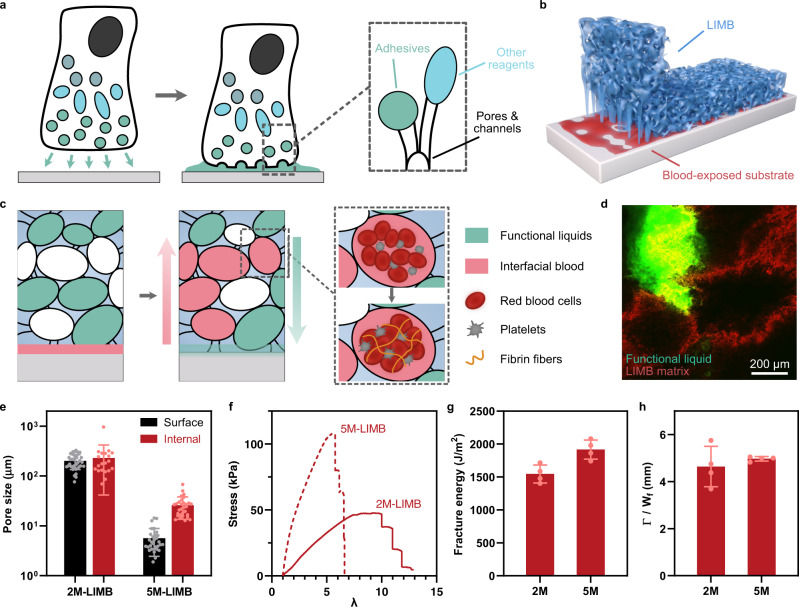
Design of LIMB. **a** Schematic illustration of marine organisms that contain interconnected micropores for adhesiveness and transport of liquid reagents. **b** Schematic of LIMB adhering on blood-exposed substrates. **c** Schematics showing that LIMB can uptake interfacial fluid, secrete functional liquids, and coagulate blood, thereby providing adhesion, hemostatic, and sealing function. **d** Confocal image of rhodamine-labeled LIMB (red) containing micropores, partially filled with a FITC-labeled chitosan functional liquid (green). **e** Sizes of surface and internal pores in LIMB containing 2 M or 5 M PAAm. **f**–**h** Stress-stretch curves (**f**), fracture energy (**g**), and fractocohesive lengths (**h**) of LIMBs with varying PAAm content. Values in **e**, **g**, **h** represent the mean ± s.d. (*n* = 40 for 2M-LIMB Surface; 20 for 2M-LIMB Internal; and 30 for 5M-LIMB Surface and Internal in **e**; *n* = 4 in **g**, **h**; The experiment was repeated three times independently with similar results for **d**).

In nature, some marine organisms adhere to bio-fouled surfaces with adhesives that feature microstructural architecture and infused liquid. Examples include mussel plaques with microporous structure^14^ and flatworms with gland channels for storage and delivery of adhesive liquids (Fig. 1a)^15^. These microstructured bioadhesives contrast with clinically used bioadhesives such as cyanoacrylate, fibrin glues, and hydrogel-based bioadhesives, which lack porous structures and infiltrated liquid^12,16^. Catechol-based adhesives, inspired by mussels, form modest wet adhesion but do not mimic the porous structures either^14^. Those non-structured/homogenous designs could avoid leakage and benefit sealing, but in turn, limit the ability to absorb and manipulate the interfacial fluid. Such a limitation is detrimental under hemorrhage conditions, as rapid pressurized blood can wash out hemostatic agents and disrupt any poorly-formed blood clots that are intrinsically brittle^17–19^. Although interfacial fluids inhibit the adhesion of materials, non-structured bioadhesives cannot rapidly remove those fluids due to the slow diffusion process and large blood components, even if a dry matrix and/or a hydrophobic repelling liquid is used^8,20^. Absorbing and resisting pressurized blood flows is thus mission-critical for hemostatic technologies in treating non-compressible hemorrhage.

Inspired by the microstructured adhesives in nature, here we propose sealants based on liquid-infused microstructured bioadhesives (LIMBs) to halt non-compressible hemorrhage. Such bioadhesives can rapidly absorb and clot whole blood while forming strong bioadhesion, without the need for compression, to resist blood pressure and seal bleeding sites (Fig. 1b-d). They are formed by infusing a macroporous hemostatic xerogel with functional liquids, different from existing non-structured bioadhesives (NBs). The hemostatic xerogel is tough, biodegradable, and active in promoting blood coagulation; its macropores potentiate rapid convection and efficient removal of interfacial fluids, such as whole blood. The infused functional liquids enable universal adhesiveness, antibacterial function, storage stability, and easy implementation. We demonstrate a significantly improved hemostatic efficacy and biocompatibility of LIMB in rats and pigs compared to clinically used counterparts. LIMB can also be instantly removed on-demand without causing rebleeding.

## Results and discussion

### Design and synthesis of LIMB

The design criteria of LIMB include: (i) The matrix should contain macropores (~100 µm) that exceed the dimensions of blood components like red blood cells (6–8 µm); (ii) The matrix should be tough to tolerate the pores and dry to imbibe the interfacial fluid spontaneously; (iii) The infused liquid should facilitate strong interfacial bonding for bioadhesion and remain stable within the matrix for repetitive usage and storage. Following these design criteria, we synthesize and test a macroporous tough xerogel as the LIMB matrix. The model xerogel is formed with covalently cross-linked polyacrylamide (PAAm) and physically cross-linked chitosan^21^, using freeze-drying; note that the PAAm is crosslinked with gelatin methacrylate (GelMA) that is enzymatically degradable^22–24^. The PAAm provides a stretchy network while physically crosslinked chitosan can dissipate energy under deformation. The xerogel after lyophilization is dry and partially infused with an adhesive functional liquid, comprising chitosan, N-(3-Dimethylaminopropyl)-N′-ethylcarbodiimide (EDC), and N-hydroxysuccin-imide (NHS), to facilitate amide bond formation with tissues (Supplementary Fig. 1). Depending on the needs of specific applications, the infused liquid can also contain other functional components, for instance, an antibacterial agent to avoid bacterial infection. The products are immediately deployed or stored at −80 °C before usage.

To meet the first design criterion, we engineer the microstructure of LIMB by optimizing the polymer concentration and gelation condition. Although fixing chitosan at 1.5% w/v, we vary the PAAm concentrations from 0.5 M (2.1% w/v) to 5 M (21% w/v); the resulting products are denoted as “*x*M-LIMB” according to *x* M PAAm concentration. Scanning electron microscopy (SEM) imaging reveals the surfaces and internal structures of the resulting xerogels, confirming the presence of interconnected macropores within LIMB after −20 °C freezing and lyophilization (Supplementary Fig. 2a). The pore size of 2M-LIMB is ~200 µm and 10- to 50-fold larger than that of 5M-LIMB (Fig. 1e), indicating an inverse relationship between the PAAm concentration and the pore size. The porous structure of 2M-LIMB is uniform throughout the matrix, whereas 5M-LIMB contains much smaller surface pores than those in the bulk (Fig. 1e). Noteworthy, the cross-section view shows nonhomogeneous phase separation in the center of 5M-LIMB (Supplementary Fig. 2b). These findings are attributed to the high stiffness and solid content of 5M-LIMB and the fast cooling at the surface, limiting the growth of ice crystals and the resulting pore size.

### Tough and pore-insensitive matrix

Macroporous structures are often vulnerable to rupture but could be circumvented by a tough and pore-insensitive matrix^25,26^. To test this point, we perform pure-shear tests to characterize the toughness and pore sensitivity of LIMB. After equilibrium in phosphate-buffered saline (PBS), LIMB exhibits high fracture energy >1500 J m^−2^ and large deformability (stretch limit >6) (Fig. 1f, g and Supplementary Fig. 3). The high toughness is also confirmed with large hysteresis loops under cyclic tensile tests up to 210% strains (Supplementary Fig. 4). The dissipative property maintains even when LIMB is partially dehydrated. These properties exceed soft tissues/organs such as cartilage and blood vessels, as well as the fully swollen tough adhesive in prior works^12,27,28^. The mechanical performance of the xerogel is attributed to its double-network design, where hydrogen bonds dissipate substantial energy and resist swelling^21,25^.

To further quantify the sensitivity to pores as defects, we estimate the critical length of flaw sensitivity by dividing the fracture toughness (Γ) by the work to fracture (${W}_{f}$, the area beneath the nominal stress-stretch curve)^29^. The critical length of LIMB is ~5 mm (Fig. 1h), over one order of magnitude larger than the size of built-in macropores. The results imply that the LIMB is immune to macropores and fracture, meeting the second design criterion.

Another important mechanical property is stiffness, which determines the conformity of LIMB to tissue surfaces. As sensitive to hydration, xerogels are infused with different amounts of adhesive functional liquid to form LIMBs, which are tested for Young’s moduli. With 25% hydration (i.e., 25% volume of LIMB is the infused liquid), 2M-LIMB exhibits much lower Young’s moduli (20-70 kPa) than that of 5M-LIMB (100-200 kPa) (Supplementary Fig. 4). According to Dahlquist criterion that soft materials are tackier when the modulus is below 100 kPa^30^, we choose 2M-LIMB as the default configuration for further investigation.

### Interfacial fluid uptake

**Fig. 2:**
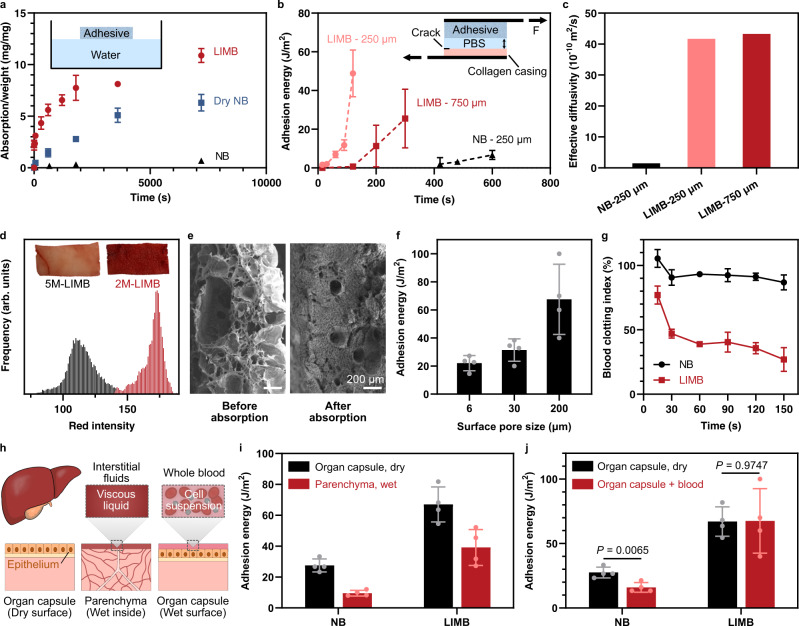
Rapid uptake of interfacial fluids and adhesion on bio-fouled surfaces. **a** Fluid absorption kinetics; **b** Adhesion formation kinetics. **c** Effective diffusivity of NB and LIMB. **d** Digital photos and red-signal distribution showing LIMBs with different surface pore size after absorbing whole blood (inset photos). **e** SEM images showing LIMB before and after absorbing whole blood. **f** Adhesion energy on blood-exposure surfaces as a function of the surface pore size. **g** Clotting kinetics of whole blood placed on LIMB and NB. The lower the index value, the higher the degree of blood clotting. **h** Schematics showing different wetting scenarios of liver surface and inside. **i** Adhesion energy of NB and LIMB on dry liver surface (capsule) and wet liver inside (parenchyma) without applying pressure. **j** Adhesion energy on blood-exposed liver surfaces without applying pressure. Values in **a**, **b**, **f**, **g**, **i**, **j** represent the mean ± s.d. (*n* = 3 for **a**, **b**; *n* = 4 for **f**, **i**, **j**; *n* = 6 for NB and 4 for LIMB in **g**; The experiment was repeated four times independently with similar results for **e**). Statistical significance and *P* values were determined by two-sided *t*-test.

The macroporous and dehydrated nature of LIMB could enable and accelerate the uptake of interfacial fluid. We characterize the speed and capacity of interfacial fluid absorption of LIMB and NBs. LIMB absorbs liquid rapidly due to the capillary suction from the large pores compared to the diffusion-dominated mechanism of NBs at either dry or wet states (Fig. 2a). The correlation between the pore size and fluid uptake can be understood according to Washburn law^11^. The time for LIMB to imbibe the interfacial fluid is given by:

1 $$t={\left(\frac{h}{\varPhi }\right)}^{2}\left(\frac{2\eta }{\gamma R\,{{\cos}}\theta }\right)$$

where $h$ is the thickness of the fluid layer, $\eta$ is the viscosity of the fluid, $\gamma$ is the fluid surface tension, $\theta$ is the contact angle, $\varPhi$ and $R$ are the porosity and pore size of the matrix, respectively. As our measurements inform $\varPhi \propto {R}^{1/5}$ (Supplementary Fig. 5), the time to absorb the fluid thus scales with ${R}^{-7/5}$. The indication that the liquid uptake accelerates with larger pores agrees well with our results.

To further benchmark the effect of interfacial fluid on adhesion, we measure the adhesion energy of LIMB on collagen casing while varying the thickness of PBS at the interface. The collagen casing mimics the tissue surface and allows precise control over interfacial fluid to match the thickness of mucus for physiological relevance^31^. We find that the time to initiate adhesion (${t}_{{{{{{\rm{ad}}}}}}}$) is within 15 s for LIMB, given the thickness of interfacial fluid (${H}_{{{{{{\rm{if}}}}}}}$) of ~250 μm; when ${H}_{{{{{{\rm{if}}}}}}}$ increases to ~750 μm, ${t}_{{{{{{\rm{ad}}}}}}}$ is prolonged to 130 s (Fig. 2b). By contrast, NB forms no adhesion within 420 s. As the adhesion is initiated upon the clean-up of the interfacial fluid, we can estimate the effective diffusivity with

2 $$D={H}_{{{{{{\rm{if}}}}}}}^{2}/{t}_{{{{{{\rm{ad}}}}}}}$$

The diffusivity of LIMB is ~4.2$\times$10^−9^ m^2^/s, which is 30-fold higher than that of NB (Fig. 2c).

### LIMB absorbs and coagulates blood

As whole blood differs from other body fluids in terms of blood cells and coagulation ability, we study the physical and hemostatic interactions between LIMB and blood. We first examine the blood absorption ability as a function of the surface pore size. As expected, 2M-LIMB with larger pores absorbs the whole blood, whereas 5M-LIMB absorbs only plasma due to steric interactions between small pores and blood cells. This finding is supported by the comparison of the red signal, an indicator of red blood cells (RBCs), between the two tested conditions (Fig. 2d). SEM imaging reveals that RBCs are found in 2M-LIMB throughout the depth of the porous matrix (Fig. 2e and Supplementary Fig. 6). A downstream effect of the blood absorption is confirmed by the fact that 2M-LIMB adheres better to the liver (Fig. 2f). These results affirm our hypothesis that large open pores can effectively absorb the blood cells and promote bioadhesion.

Another advantage of LIMB is its ability to promote blood coagulation near and within the LIMB matrix. This clotting ability is quantified with a blood clotting index, reflecting the amount of free RBCs that are not within clots, following a previously reported protocol^2,32–34^. The blood clotting index correlates inversely with the degree of blood clot formation. We find that the clotting occurs within seconds upon the contact between LIMB and whole blood (Fig. 2g). This phenomenon is unseen in NB, despite the presence of the same chitosan polymer with known hemostatic function. We thus contribute the difference to the porous and dehydrated nature of the LIMB matrix, which contacts and concentrates RBCs and platelets in the absorbed blood, and thus accelerates the clotting cascade substantially^35^. Besides hemostasis, the clot formation could help obstruct the pores within LIMB to avoid transmembrane leakage for sealing performance, as shown later. The clotting ability differentiates LIMB from the bioadhesive sealants reliant solely on physical barrier effects in prior works^7,8^.

### Tough bioadhesion on bio-fouled surfaces

Many tissue surfaces are fouled/covered with biological substances such as mucus and blood, which impair the performance of bioadhesives. Take the liver as an example; the outer part, Glisson’s capsule, may be covered with blood under traumatic and surgical conditions, while its inner part, parenchyma, is layered with a viscous interstitial fluid (Fig. 2h). We test the bioadhesion performance of LIMB on these surfaces without compression and compare it with NB (Supplementary Fig. 7). On the dry organ capsule surface, the adhesion energy of LIMB is 2.4 times higher than that of NB (Fig. 2i). Higher contrast is found on the wet parenchyma, where the adhesion energy of ~40 J m^−2^ for LIMB is more than 4 times higher than ~9 J m^−2^ for NB. Note that the weaker adhesion on parenchyma than on the organ capsule is likely because the parenchyma is more fragile. A similar conclusion stands when the surfaces are fouled with blood. The performance of NB deteriorates as the blood components impair the contact between NB and the tissue. No such reduction is observed with LIMB since it can effectively absorb and remove the blood as shown above (Fig. 2j).

### Pressure-insensitive adhesion to diverse materials

**Fig. 3:**
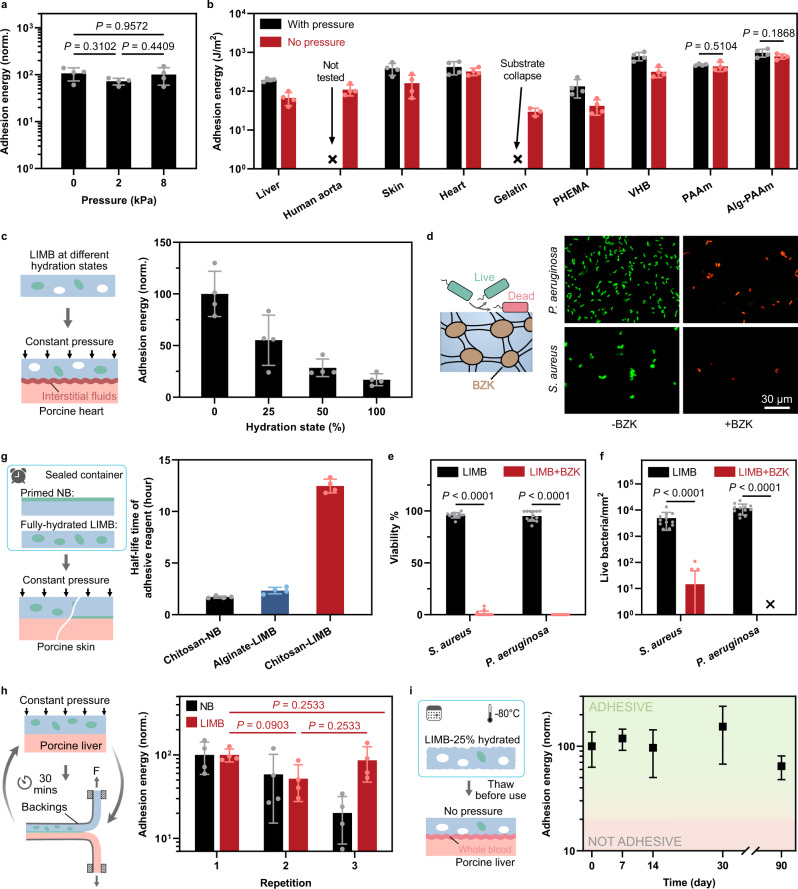
Pressure-insensitive adhesion and effects of infused liquid on adhesion, antibacterial function, and usability. **a** LIMB is pressure-insensitive at a low-pressure range. **b** Adhesion performance on diverse substrates with and without applying pressure (~60 kPa) to form initial adhesion. The adhesion on the human aorta with pressure was not measured due to the unavailability of tissue samples. **c** Adhesion energy as a function of hydration state. **d** Benzalkonium chloride (BZK) as an antimicrobial functional liquid can enable LIMB with antimicrobial function, as evidenced by the LIVE/DEAD assays on *P. aeruginosa* and *S. aureus*. **e** Viability and **f** Live cell attachment comparisons between LIMB and LIMB loaded with BZK. **g** EDC half-life time when stored in different adhesives. **h** Infused liquid has a reservoir effect which enables repeatable adhesion. **i** Infused liquid can be stored within LIMB at low temperature for prolonged shelf life. Values in **a**–**c**, **e**–**i** represent the mean ± s.d. (*n* = 4 for **a**–**c**, **g**–**i**; *n* = 12 for **e**, **f**). Statistical significance and *P* values were determined by two-sided *t*-test for the comparison between two groups and one-way ANOVA with post hoc Tukey tests for comparison between multiple groups.

Besides liver, LIMB can achieve strong and universal adhesion without compression on diverse surfaces. For instance, the adhesion between LIMB and porcine skin is independent of applied pressure (0-8 kPa) (Fig. 3a). Similar adhesion performance is found on other tissues, such as the human aorta and porcine heart, with or without pressure (Fig. 3b). As a demonstration, LIMB forms stable adhesion to a blood-exposed porcine heart without the need for compression (Supplementary Fig. 8 and Movie 1). In addition to tissues, LIMB exhibits pressure-insensitive adhesion on hydrogels and elastomers (Fig. 3b). Without the pressure, LIMB can achieve a strong adhesion that outperforms fibrin glues and medical tapes (10–20 J m^−2^)^21,27^. Among the tested hydrogels, gelatin hydrogels are too fragile to survive the compression for adhesion formation. The robust pressure-insensitive adhesion is attributed to the unique structure of LIMB: the xerogel matrix can spontaneously absorb interfacial fluids, forming intimate contact and strong bonding with the substrates with the aid of the infused liquid. This feature enables a broad utility of LIMB in diverse medical and engineering settings.

### Tunable functionality with infused liquid

The performance and functionality of LIMB are tunable with the infused liquid. As shown above that the uptake of interfacial fluids influences adhesion, we hypothesize the amount of infused liquid or the hydration state could mediate the adhesion performance of LIMB. We load and test LIMB with different amounts of the adhesive functional liquid (0–100%) on wet porcine heart (Fig. 3c). For 0%-hydrated LIMB, the surface of initially dry LIMB is primed with the adhesive functional liquid and immediately applied onto the tissue. As expected, the adhesion energy scales inversely with the hydration state (Fig. 3c). A similar correlation is found between the adhesion formation speed and the hydration state, where 0%-hydrated LIMB forms instant adhesion with wet tissues upon contact while 100%-hydrated LIMB does not (Supplementary Fig. 9). The viscosity of the adhesive functional liquid seems to impair the adhesion performance, but its role is less significant compared with the loading amount of the functional liquid (Supplementary Fig. 10).

The infused liquid can also functionalize LIMB. We demonstrate this possibility by loading LIMB with an antimicrobial agent, 5% benzalkonium chloride (BZK), for the antibacterial function that is desired in many surgical settings^36^ (Fig. 3d). We test the antimicrobial and antifouling functions by exposing BZK-laden LIMB to two model pathogenic bacteria: *P. aeruginosa* and *S. aureus*. Compared with the pristine LIMB, BZK-LIMB exhibits more prominent bacteria-killing and attachment inhibition effects (Fig. 3e, f). The results demonstrate the tunability and modularity of LIMB with the liquid-infiltration design.

### Ease of use and stability of long-term storage

The unique design of LIMB also helps address important translational considerations about usability and storage. Minimizing chemical reactions between the liquid and the matrix is a premise for extending the time window for usage and prolonging the stability of LIMB. To this end, both the matrix and the infused liquid of LIMB are chosen based on chitosan (primary amine-rich); as such, no carbodiimide reaction would occur. To test this point, we also prepare another LIMB made from alginate (carboxylic acid-rich) for comparison. The chitosan- and alginate-based LIMB are loaded with the same adhesive functional liquid (chitosan, EDC, and NHS) and stored at 4 °C in sealed containers. We characterize the adhesion energy of LIMBs on porcine skin as a function of storage duration. The chitosan-based LIMB maintains adhesiveness even after 24 h (Supplementary Fig. 11). Increasing storage temperature tends to lower the stability of LIMB. The adhesion energy of LIMB stored at room temperature decreases with the storage duration, which can be linked with the hydrolysis of adhesive agents such as EDC. Nevertheless, the stored LIMB maintains appreciable adhesiveness for 6 h (Supplementary Fig. 12), which satisfies most surgical procedures. In comparison, the alginate-based LIMB exhibits a much shorter half-life time, as the infused liquid reacts with the alginate-based matrix via carbodiimide reactions (Fig. 3g); for NB, the loss of adhesiveness is attributed to the shortage of adhesive reagents.

With the confirmed compatibility, LIMB is anticipated to maintain adhesiveness during repositioning and long-term storage. On the one hand, the adhesive agents stored in macropores can replenish the surface for repeatable adhesion. We measure the adhesion energy by attaching the same 50%-hydrated LIMB to fresh porcine livers three successive times, mimicking the repositioning scenario. LIMB maintains appreciable adhesion (>30 J m^−2^) in all repetitions. In contrast, the adhesion energy of NB at the third attempt decreases to one-sixth of that at the first attempt (Fig. 3h). Such a salient feature allows the corrections of the location of LIMB for optimal placement. On the other hand, LIMB could be stored for an extended period at −80 °C, which is commonly used for the storage of therapeutics and chemicals. The low temperature can inhibit the degradation of the adhesive agents and further improve their stability within LIMB. To test this possibility, we examine the adhesion performance of 25%-hydrated LIMB after storage on blood-exposed liver capsules without applying compression and find that LIMB keeps highly adhesive over 90 days (Fig. 3i). These collective attributes support the convenience and usability of LIMB.

### In vitro swelling, biodegradation, and cytocompatibility

**Fig. 4:**
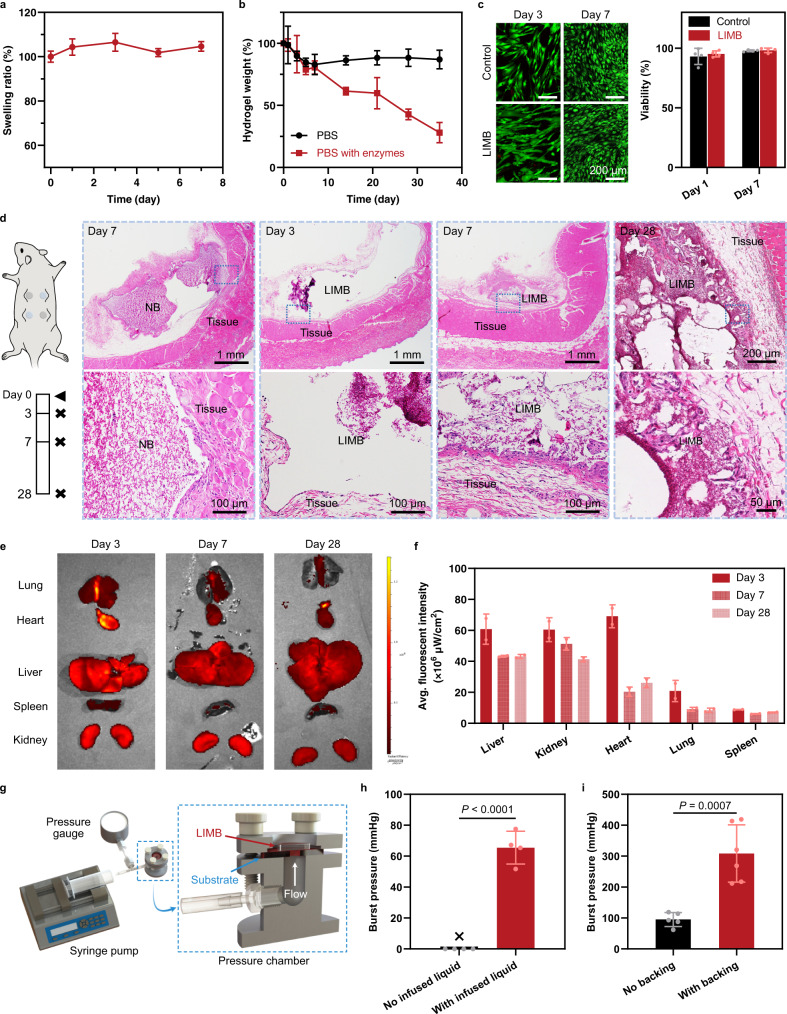
Evaluation of swelling, degradability, biocompatibility, and sealing performance. **a** Swelling of LIMB in PBS. **b** Remaining hydrogel weight over time when the hydrogels are exposed to PBS and PBS with enzymes (collagenase and lysozyme). **c** LIVE/DEAD assay of cells in control and LIMB. Control: cell culture on a standard 96-well plate. **d** Schematic and histological images showing the implants and the nearby tissues after 3-, 7-, and 28-day post-implantation. LIMBs used here were 2M-LIMB with 25% of the volume infused with adhesive functional liquid. **e** Biodistribution of degraded products from LIMB in major organs over a 28-day period. FITC-labeled LIMBs were used to show fluorescence. **f** Average fluorescent intensity over time from biodistribution. **e** Schematic showing burst pressure measurement. **f** Burst pressure of LIMB with and without infused liquid. LIMB without infused liquid does not exhibit a noticeable sealing function while LIMB with infused liquid resists the flow to pass through at reasonable burst pressure. **g** Burst pressure of 25%-hydrated LIMB with and without a stiff backing. Values in **a**–**c**, **f**, **h**, **i** represent the mean ± s.d. (*n* = 4 for **a**–**c**, **h**; *n* = 2 biologically independent animals for **e**, **f**; *n* = 5 for No infused liquid and 6 for With infused liquid in **i**; The experiment was repeated four times independently with similar results for **d**). Statistical significance and *P* values were determined by two-sided t-test.

We combine a series of in vitro and in vivo tests to evaluate the safety and efficacy of LIMB for hemorrhage control. Although LIMB contains hydrophilic PAAm, the physically cross-linked chitosan network can effectively suppress the swelling of the hybrid network. As a result, LIMB exhibits almost no swelling when immersed in PBS (Fig. 4a and Supplementary Fig. 13). The incorporation of a degradable cross-linker for the PAAm network and the biodegradable chitosan renders LIMB biodegradable. A steady degradation profile of LIMB is observed when exposed to an enzyme solution comprising lysozyme and collagenase at physiological levels (Fig. 4b). The biodegradability is essential for hemostatic use to avoid the need for removal and secondary surgeries^23,37^. We also evaluate the cytocompatibility of LIMB according to the International Organization for Standardization (ISO) 10993-5: Tests for in vitro cytotoxicity standard. Immortalized human vocal fold fibroblasts (hVFFs) are used. The in vitro cell culture shows over 98% cell viability throughout a 7-day culture, proving the cytocompatibility of LIMB (Fig. 4c).

### In vivo biocompatibility and biodegradation

The in vivo biocompatibility of the LIMB is examined via subcutaneous implantation in rats for 28 days and compared with NB. The explants maintain physical integrity after the implantation (Supplementary Fig. 14 and Fig. 15). Histological sections show a very thin capsule with rare foreign body-type giant cells at the interface between the tissues and all the implants (Fig. 4d); there is no evidence of active or chronic inflammation or allergic eosinophilic responses. Consistent with the cytocompatibility test, the results conclude that LIMB and NB elicit comparable minimum inflammation responses in vivo. The toxicity of EDC is dependent on concentration; when used at low concentrations, EDC is found to induce no severe toxicity^23,27,38–40^ (Supplementary Table 1). Considering the strong adhesion and sealing effect of EDC, its benefits in treating life-threatening situations outweigh its local cytotoxicity. It is also worth noting that EDC could be replaced by other bioadhesive agents, such as transglutaminase^41^, oxidized polysaccharides^42^, and catechol-modified biopolymers^43^, which are considered biocompatible and capable of bonding LIMB with biological tissues. LIMB as a platform technology can be customized to accommodate a variety of adhesive agents.

We also investigate the in vivo biodegradation and evaluate the toxicity of the degradation products. By using FITC-labeled LIMBs and IVIS biodistribution imaging, we map out the clearance pathway of LIMB over 28 days (Fig. 4e). As expected, the clearance is mainly through the liver and kidneys (Fig. 4f). A slow and steady change in size is also observed (Supplementary Fig. 15). Upon histological analysis, there is no sign of systematic toxicity to any major organs at Day 28 (Supplementary Fig. 16), suggesting the safety of LIMB for long-term implantation.

### In vitro sealing test

The combination of hemostatic and bioadhesion performance allows LIMB to seal the lateral and transmembrane flows of body fluids. Although the macroporous structure concerns leakage, the infiltration of the adhesive functional liquid within LIMB could provide liquid gating effects to ensure sealing^44,45^. To prove this point, we test the burst pressure of LIMB as a function of liquid infiltration. When 25% of the pores are infused with liquid, the 25%-LIMB exhibits high burst pressure at ~66 mmHg, in contrast to almost no sealing from LIMB without infused liquid (Fig. 4g, h). The decent sealing is attributed to the infused liquid that closes the macropores for blood leakage. When tested on heart tissues, the 25%-LIMB achieves ~95 and ~308 mmHg of burst pressure with and without rigid backing support, respectively, showing an excellent sealing performance (Fig. 4i). The sealing is prolonged as shown that the attachment to the tissue persists even after being immersed under PBS for over 8 days (Supplementary Movie 2).

### In vivo hemostatic tests

**Fig. 5:**
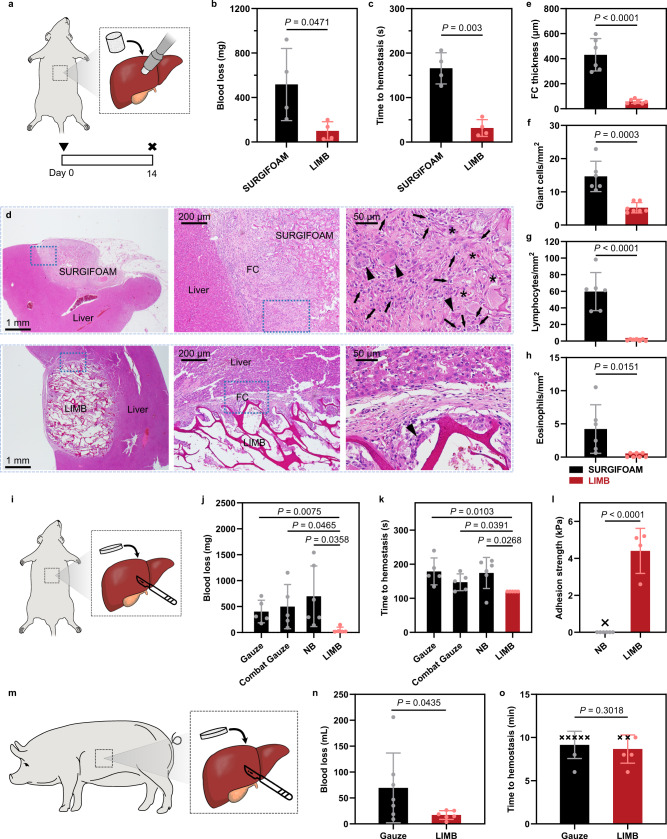
In vivo validation of the safety and efficacy for hemorrhage control. **a** Illustration of a liver volumetric defect non-compressible hemorrhage model on rat without applying pressure. **b** Blood loss and **c** time to hemostasis comparison. **d** Histology images showing the implants after 2-weeks. “Triangle” symbols point to foreign-body type giant cells; “Arrow” symbols point to lymphocytes; “Star” symbols point to eosinophils. **e**–**h** Immune responses comparisons. **i** Illustration of deep incision hemorrhage on rat liver. **j**, Blood loss and **k**, time to hemostasis comparisons among different hemostats. **l** Adhesion strength comparison after hemostasis at ~120 s. **m** Illustration of a deep incision hemorrhage on pig liver. **n** Blood loss and **o**, time to hemostasis comparisons. Values in **b**, **c**, **e**–**h**, **i**–**l**, **n**, **o** represent the mean ± s.d. (*n* = 4 for **b**, **c**; *n* = 6 for SURGIFOAM and 7 for LIMB in **e**–**h**; *n* = 5 for Gauze, Combat Gauze, and LIMB and 6 for NB in **j**, **k**; *n* = 6 for NB and 4 for LIMB in **l**; *n* = 7 for Gauze and 6 for LIMB in **n, o**; independent samples). Statistical significance and *P* values were determined by two-sided *t*-test for **b**, **c**, **e**–**h**, **i**–**l** and one-sided *t*-test for **n**, **o**.

We evaluate the safety and efficacy of LIMB in different animal models with physiological relevance in comparison with commercial absorbable hemostatic gelatin sponge SURGIFOAM (Ethicon). Deep wounds with small entrances, for instance, caused by small arms fire limit the direct contact between hemostats and bleeding vessels^46^. The resulting non-compressible hemorrhage has limited treatment outcomes with current hemostatic technologies^2,32^. We use a rat liver volume defect model (4 mm in diameter and 3 mm in depth) to evaluate the efficacy of LIMB as an implantable hemostat to halt non-compressible hemorrhage (Fig. 5a and Supplementary Fig. 17). The liver defect receives LIMB or SURGIFOAM of the same size. The hemostatic efficacy of LIMB is manifested with low blood loss (99.8 mg) and quick time to hemostasis (31.5 s). Compared with 517.1 mg and 165.8 s for SURGIFOAM, our material achieves an overall 5-fold improvement in both criteria (Fig. 5b, c).

In addition to the acute response, long-term biocompatibility in vivo is evaluated by examining the materials implanted for 2 weeks after hemostasis (Fig. 5d). Four metrics of potential immune responses are used, including the thickness of fibrotic capsule (FC), giant cells density for foreign body reactions, lymphocytes density for inflammatory responses, and eosinophils density for allergy responses. Statistical analysis substantiates a considerable advantage of LIMB over SURGIFOAM in all metrics measured. More specifically, LIMB induces a thin FC (~53 µm), very mild foreign body reactions, minimal inflammation, and allergy responses. In comparison, SURGIFOAM leads to a thick FC (~431 µm) and a high degree of foreign body and inflammation responses; the allergy response is moderate but significantly higher than that of LIMB (Fig. 5e-h). Overall, LIMB demonstrates great promise to be used as implantable hemostats for halting non-compressible hemorrhages.

We further test LIMB for treating deep incision hemorrhage. Both rat and pig liver incision models are used; NB, standard gauze, and a commercially available Combat Gauze (QuickClot) are included for comparison. The hemostats are positioned steadily at bleeding sites and exposed to no pressure to test the pressure-insensitive hemostasis^43^. For the rat model, an incision of 7 mm in length and 3 mm in depth is created on the liver. The resulting blood loss is significantly lower for LIMB compared with other hemostats, including NB, standard gauze, and Combat Gauze (Fig. 5i, j and Supplementary Fig. 18). Owing to the translucent appearance, the exact time to hemostasis of LIMB is difficult to measure. All the tested LIMBs stop the bleeding within 120 s’ check-up point and are much quicker than other products (Fig. 5k). We use a “pull-off” test to measure the adhesion strength of LIMB to the bleeding liver at ~120 s, before the formation of chemical bonds. LIMB is securely attached to the wounded liver based on capillary suction alone, showing a significantly higher adhesion strength than NB (Fig. 5l). The formation of adhesion indicates that LIMB successfully removed the interfacial blood. For the pig model, an incision of 15 mm in length and 10 mm in depth on the liver is created (Fig. 5m and Supplementary Fig. 19). The average blood loss for gauze and LIMB are 69.5 and 17.1 mL, respectively (Fig. 5n). We also observe the time to hemostasis within a 10-minute cut-off time for both groups. Successful hemostasis events are denoted with dots and unsuccessful hemostasis with crosses (Fig. 5o); 4 out of 6 swine injuries treated with LIMB stop bleeding within 10 min, but only 2 out of 7 injuries treated with plain gauze succeed. The successful hemostasis rate is 66.7% for LIMB and 28.6% for gauze, an over 2.3-fold improvement. The combined results prove the efficacy of LIMB in halting deep incision hemorrhages.

### LIMB removal

LIMB can be instantly and safely removed on-demand, which is an unmet need for existing bioadhesives. The adhesion of LIMB contains two stages. In the first stage, roughly the first 2 min following the placement, the adhesion mainly comes from the capillary suction from the dry pores of LIMB. These physical interactions can be easily disabled by wetting LIMB with saline (0.9% NaCl). We show that LIMB can be safely peeled off from the wounded liver immediately after wetting (Supplementary Fig. 20 and Movie 3). Similarly, this method applies to repositioning procedures as they are usually decided within 1–2 min. LIMB can be detached from the surface easily with the wetting or be directly peeled off.

Beyond this time window, LIMB is still removable with removal agents to cleave bonds formed at the interface. The removal agents are needed because covalent bonds appear after 2 min due to EDC, and plateau in ~10 min. Once chemical bonds form, LIMB cannot be easily detached from the tissue simply by wetting (Supplementary Movie 4). We demonstrate two removal agents: acetic acid solution (0.1 M) and lysozyme solution (75 mg/mL). Acetic acid can quickly disrupt those bonds and even dissolve chitosan networks at the interface and within LIMB, which are responsible for wet adhesion (Supplementary Movie 5); note that the acetic acid solution used is at a low concentration and can be quickly neutralized with saline afterward. Alternatively, lysozyme acts like scissors to cut down the chitosan chains. After 10-min exposure to lysozyme, the adhesion between LIMB and the wounded liver is noticeably weakened (Supplementary Movie 6). Noteworthy, we observed no rebleeding after the removal of LIMB with either acetic acid or lysozyme. In vitro experiment also confirmed the effectiveness of removal agents for on-demand LIMB removal (Supplementary Fig. 21). We attribute this phenomenon to the capacity of LIMB to promote coagulation, an advantage over existing bioadhesive sealants that only physically block the bleeding site.

In this article, we have described a microstructured hemostatic bioadhesive design by leveraging the bioinspired architecture, tough adhesives, and liquid infiltration. The resulting microstructured bioadhesives can form instant and strong adhesion with bio-fouled surfaces without the need for compression. The bioadhesives are biodegradable, easy to implement, and stable for long-term storage. The functionality of the adhesives is readily tunable with the infused functional liquid. Their biocompatibility and hemostatic efficacy outperform several existing hemostatic agents and bioadhesives, as demonstrated by the decreased blood loss in non-compressible and deep-and-narrow hemorrhages in small and large animal models. They are also instantly and safely removable on-demand. We anticipate that the reported design principle and the material system would galvanize the development and translation of bioadhesives and hemostatic materials to manage hemorrhage.

## Methods

### Materials

All chemicals were purchased and used without further purification. The materials for hydrogel synthesis include: acrylamide (AAm, Sigma, A9099), N,N′-methylenebisacrylamide (MBAA; Sigma-Aldrich, M7279), ammonium persulfate (APS, Sigma-Aldrich, A3678), tetramethylethylenediamine (TEMED, Sigma-Aldrich, T7024), chitosan (degree of deacetylation, DDA: 95%, medium and high molecular weight, Lyphar Biotech), alginate (high molecular weight, I-1G, KIMICA Corporation), sodium bicarbonate (Fisher Scientific, S233), sodium phosphate monobasic (NaH_2_PO_4_, Sigma-Aldrich, S8282), sodium phosphate dibasic (Na_2_HPO_4_, Sigma-Aldrich, S7907), acetic acid (Sigma-Aldrich, A6283), benzalkonium chloride (BZK, Fisher Scientific, AA4133914). Gelatin methacrylate (GelMA) was synthesized according to a previously reported protocol^47^ and used as a degradable crosslinker. Materials for adhesion experiments include: N-(3-Dimethylaminopropyl)-N′-ethylcarbodiimide hydrochloride (EDC, Sigma-Aldrich, 03450), N-hydroxysuccinimide (NHS, Sigma-Aldrich, 130672), collagen casing (Weston), and VHB (3 M). Porcine liver, heart, and skin tissues were purchased from a local grocery store. Materials for synthesizing fluorescently labeled hydrogels include: fluorescein-5 isothiocyanate (Thermo Fisher, F1907), rhodamine-B isothiocyanate (Cayman Chemical, 20653), anhydrous methanol (Fisher Scientific, A412-1), 0.22 µm PES filters (Fisher Scientific, 13100106), and 3.5 K MWCO dialysis tubing (Fisher Scientific, PI88244).

### Synthesis of non-structured bioadhesive (NB) matrix

Both acrylamide and chitosan powders were first dissolved in 0.2 M acetic acid at 3.3 mol/L and 2.5%, respectively. GelMA was added to the AAm-chitosan solution as degradable crosslinker at a concentration of 0.11% w/v. A gelling solution to induce physical cross-linking of chitosan was prepared by first mixing 0.1 M Na_2_HPO_4_ and 0.1 M NaH_2_PO_4_ at a volume ratio of 50:3, followed by the addition of sodium bicarbonate to a final concentration of 0.306 M. APS was added to the gelling solution at a concentration of 0.225% as initiator. Both solutions were degassed, quickly mixed at 3:2 volume ratio (precursor solution versus gelling solution), and poured into a glass mold for gelation at room temperature overnight.

### Synthesis of chitosan xerogel (LIMB matrix)

NB was first prepared based on the abovementioned protocol. To generate pores, NB was first dialyzed in deionized (DI) water for 1 day to remove unreacted reagents. 2M-xerogel was completed by first placing dialyzed NB into a −20 °C freezer for 24 h to form ice crystals and then freeze-dried. For 5M-xerogel, the same procedure was applied, except that the initial concentration of acrylamide in the precursor solution was 8.3 M. For 0.5M-xerogel, both acrylamide and chitosan powders were first dissolved in 0.2 M acetic acid at 0.83 mol/L and 2.5%, respectively. GelMA was added to the AAm-chitosan solution at 0.11% w/v. TEMED was then added to the polymer solution at a concentration of 0.5%. APS was dissolved in DI water at a concentration of 0.625% to form the initiator solution. The solutions were cooled to 4°C to slow polymerization before freezing. The solutions were then quickly mixed at 3:2 volume ratio (precursor solution versus initiator solution) and poured into a pre-cooled (−20 °C) glass mold. After an incubation period of 24 h at −20 °C, the gels were taken out from the mold and thawed in a pre-cooled (4°C) 0.306 M sodium bicarbonate solution. The gels were then first dialyzed in DI water for 1 day to remove unreacted reagents before lyophilization to form the xerogel for the use of LIMB matrix.

### Synthesis of alginate xerogel

Alginate-based xerogel was firstly prepared by dissolving acrylamide and alginate powders in DI water to reach a concentration of 2 mol/L and 2.256%, respectively. MBAA was added to the AAm-alginate solution at a weight ratio of 0.0006:1 (the weight of MBAA versus acrylamide) to form the precursor solution. A gelling solution was prepared by dissolving 6.87% CaSO_4_ and 3.58% APS in DI water. The polymer precursor and the gelling solution were then transferred into two syringes separately. The volume ratio of the precursor solution to the gelling solution was 24:1. Both solutions were degassed inside the syringes before use. TEMED was added to the degassed precursor syringe at a weight ratio of 0.0028:1 (the weight of TEMED versus acrylamide) before mixing. The two solutions were quickly mixed with a syringe connector and poured into a glass mold. The cross-linked hydrogel was then transferred to a −20°C freezer for 24 h and then lyophilized for at least 48 h to form alginate xerogel.

### Functional liquids preparation

To obtain the adhesive functional liquid, 2% chitosan was first dissolved in 0.14 M acetic acid to a final pH of 5. The solution was stirred overnight before use. EDC and NHS were then added to the chitosan solution, each at 20 mg/mL. To obtain the antibacterial functional liquid, 5% BZK were dissolved in water and stirred overnight to yield a clear solution at room temperature.

### LIMB preparation

LIMB was prepared by infusing a functional liquid into an xerogel. Specifically, the functional liquid was first applied to one side of the xerogel at room temperature. The volume of the functional liquid was controlled precisely for a certain volume fraction of the xerogel. An applicator, such as a pipette tip, was used to evenly distribute the functional liquid on the xerogel surface. The functional liquid was spontaneously absorbed into the xerogel without intervention. After 5–10 min, LIMB was ready to use or being transferred into a −80 °C freezer for long-term storage.

### Synthesis of fluorescently labeled chitosan

In all, 1 wt% chitosan was first prepared by dissolved chitosan in 80 mM acetic acid. The solution was sterilized through 0.22 mm PES filters before use. An equal volume of anhydrous methanol was added to the filtered chitosan solution and stirred for 3 h at room temperature. The mixture was degassed before use. Rhodamine B and FITC were dissolved in anhydrous methanol at 2 mg mL^−1^ and 1 mg mL^−1^, respectively. The staining solution was added to the chitosan/methanol mixture drop-by-drop under stirring. The final concentration of fluorescent dyes in the reaction medium was controlled to give the label to D-glucosamine residue at a ratio of 1:50. The reaction lasted for 18 h for rhodamine B-labeled chitosan and 1 h for FITC-labeled chitosan in the dark at room temperature. Then, a NaOH solution (1 mol/L) was used to precipitate chitosan from the solution. The precipitates were collected and dialyzed against DI water for 4 days.

### Morphology imaging and characterization

The porous structure of the samples was imaged using a field emission scanning electron microscope (F50, FEI) under various magnifications. LIMBs were coated 4 nm Pt using a high-resolution sputter coater (ACE600, Leica) to increase their surface conductivity. Pore size was analyzed by measuring at least 20 pores for each type of samples using the measuring tool in ImageJ. Porosity was calculated by first transforming the SEM images into binary images and dividing the number of white pixels by the number of black pixels. To image samples after blood absorption, the samples were first dehydrated using a CO_2_ supercritical point dryer (CPD030, Leica) to preserve the original morphology before SEM imaging.

### Fracture toughness measurement

The fracture toughness of hydrogels was measured using pure shear tests. A pair of samples (width of 80 mm, thickness of 1.5 mm) were glued to rigid acrylic clamps for each test. One sample was unnotched, and the other one was edge-notched. The height ($H$) of the specimen (i.e., the distance between the two acrylic clamps) was 5 mm. The unnotched sample was pulled by an Instron machine (Model 5965) with a 1 kN load cell at a strain rate of 2 min^−1^ to measure the stress-stretch ($S-\lambda$) curve. For the notched sample, a notch length of ~30 mm was introduced to an edge of the sample by a razor blade. The notched sample was pulled until rupture to obtain a critical stretch (${\lambda }_{{{{{{\rm{c}}}}}}}$). Following prior works^48,49^, the fracture energy was calculated from $S-\lambda$ curve of the unnotched sample by

3 $$\varGamma=H\int _{1}^{{\lambda }_{c}}{Sd}\lambda$$

### Tensile test

To measure the tensile behavior under cyclic loading, the xerogels were cut into strips of length 35 mm, width 5 mm, and thickness 1.5 mm, and tested with an Instron machine (10 N load cell). The displacement rate was 100 mm min^−1^. The nominal (engineering) stress was obtained by dividing the force by the initial cross-sectional area. The nominal (engineering) strain was obtained by dividing the change in length by the original length.

### Adhesion test

Adhesives and substrates were cut into strips of length 80 mm, width 15 mm, and thickness 1.5 mm. Rabbit whole blood of 150 μL was dispensed evenly on substrates under some circumstances. The adhesives were brought into contact with various substrates, incubated for 30 mins at either room temperature or 4 °C in a sealed container, and then tested with peeling tests using an Instron machine (10 N or 1 kN load cell). Rigid polyethylene (PET) films (100 µm in thickness) were glued to the back of the substrate and the adhesives, respectively, using superglue (Krazy Glue) before testing. For 90° peeling tests, the displacement rate was 50 mm min^−1^. The adhesion energy of the specimen was calculated by dividing the average force at the plateau (${F}_{{{{{{\rm{avg}}}}}}}$) by the width of the specimen:

4 $$\varGamma={F}_{{{{{{\rm{avg}}}}}}}/W$$

For T-peeling tests, the displacement rate was 100 mm min^−1^. The adhesion energy of the specimen was calculated by dividing two times ${F}_{{{{{{\rm{avg}}}}}}}$ by the width of the specimen:

5 $$\varGamma={2F}_{{{{{{\rm{avg}}}}}}}/W$$

For adhesion tests involving human aorta, the samples were provided by Transplant Québec after approval of McGill ethical committee. A total of 4 descending thoracic aorta samples were extracted from two organ donors. One donor was a 66-year-old male with 86 kg in weight and 162 cm in height. The cause of death was head trauma. The donor was healthy and with no disease. The other donor was a 47-year-old male with 92 kg in weight and 188 cm in height. The cause of death was stroke.

### Interfacial fluid absorption

The swelling kinetics of LIMB, NB, and dry NB (air-dried) were measured by placing the samples on the surface of a water reservoir with only one surface in contact with the liquid. Contact time was varied from 1 to 7200 s at room temperature. The water absorption per sample weight was calculated by $\frac{{m}_{1}-{m}_{0}}{{m}_{0}}$, where ${m}_{1}$ is the measured sample mass after absorption and ${m}_{0}$ the initial sample mass.

### Adhesion kinetics

The adhesion kinetics of NB and 25%-hydrated LIMB were characterized using a modified lap-shear setup. Both the adhesives and collagen casing were firstly glued to a rigid PET backing. PBS was then evenly coated to the collagen casing on a flat surface to form a liquid barrier layer. An initial crack of 1 mm was reserved at the front of the overlapping area by attaching a piece of thin parafilm to the collagen before adding PBS. The thickness of PBS was controlled by the volume of the liquid. The adhesive with backing was then brought into contact with the collagen casing covered with the liquid barrier layer without applying any pressure. The overlapping area (width × length) of the adhesive and the collagen casing was controlled to be 20 × 20 mm^2^. At a pre-determined time, the lap shear test was performed to measure the line force $F$ (loading force/width) and nominal strain $\varepsilon$ (displacement/length) curves. The adhesion energy was calculated using

6 $$G=\int _{0}^{{\varepsilon }_{{{\max }}}}{Fd}\varepsilon$$

where ${\varepsilon }_{{{\max }}}$ is the displacement at which $F$ reached the maximum.

### In vitro blood clotting assay

Clotting time assay was performed according to a previously reported protocol^2,32–34^. This assay was based on the fact that RBCs within a clot are less prone to break in hypertonic conditions compared to free RBCs due to their shape changes during clotting^50^. LIMB and NB were both prepared in cylindrical shapes with a height of 5 mm and a diameter of 10 mm. A volume of 50 μL of recalcified human whole blood (8 μL of 0.2 M CaCl_2_ added to per 100 μL of human blood, purchased from BioIVT) was added to the sample in a centrifuge tube. Each sample reacted with blood for 15, 30, 60, 90, 120, and 150 s. DI water of 10 mL was then added to stop the reaction and to dissolve hemoglobin from free RBCs. A negative control comprising 50 μL of recalcified whole blood in a centrifuge tube gave a reference value. The content of hemoglobin in the solution was measured by the absorbance of the supernatant at 540 nm using a microplate reader (Synergy HTX, Agilent). Six replicates were performed for each condition. The blood-clotting index (BCI) was calculated using the equation:

7 $${{{{{{\mathrm{BCI}}}}}}}\left(\%\right)=({I}_{s}-{I}_{0})/({I}_{r}-{I}_{0})$$

where ${I}_{s}$ is the absorbance of the sample, ${I}_{r}$ is the absorbance of the reference value (negative control), and ${I}_{0}$ is the absorbance of DI water.

### Cytocompatibility

The cytotoxicity of LIMB matrix was evaluated using immortalized human vocal fold fibroblasts, following the International Organization for Standardization (ISO) 10993-5: Tests for in vitro cytotoxicity. For every 200 mg of LIMB matrix, 1 mL of Dulbecco’s Modified Eagle’s medium (DMEM) was added for extraction. Meanwhile, 20,000 cells per well were seeded in a 96-well plate. The extracts, after a 24-hour incubation period, were collected and supplemented with 1% nonessential amino acid, 1% penicillin-streptomycin, and 10% fetal bovine serum. The culture medium within the 96-well plate was then replaced with the supplemented LIMB matrix extracts. Completed pristine DMEM was used as the control. The cells were then cultured for 24 h inside an incubator, in an environment at 37 °C, 95% relative humidity, and 5% CO_2_ atmosphere. Cell viability was assessed using a Live/Dead viability kit (Invitrogen, L3224), following the protocol of the manufacturer. Confocal laser scanning microscopy (Zeiss, LSM710) was used for investigation. Live cells were visualized in green and dead cells in red.

### In vitro biodegradation

All LIMB samples were prepared of the same size and weighed at Day 0. After that, an enzyme solution consisting of 50 µg/mL collagenase (MP Biomedicals, 1951091), 50 µg/mL lysozyme (MP Biomedicals, 100831), and 0.01% sodium azide (NaN_3_, Sigma, S2002) in PBS was added to the gels. The samples were incubated at 37 °C with gentle mechanical stimulation at 75 rpm over 35 days. The enzyme solution was changed every other day. At pre-determined time intervals, the enzyme solution was removed. The samples were then washed three times (5 min each wash) with deionized water. The samples were then lyophilized, and the remaining polymer dry weight was measured. The remaining ratio of the polymer was calculated by dividing the remaining polymer dry weight by the initial dry weight of gels.

### In vitro swelling

LIMBs were first prepared into disk shapes (5 mm in diameter and 1.5 mm in height) and then fully immersed inside PBS in a sealed Petri dish. The samples were incubated at 37 °C with gentle mechanical stimulation at 75 rpm over 7 days. The swelling ratio was calculated by dividing the measured diameter by the diameter of the initial gels at the dry state.

### Storage stability

LIMBs were first prepared by infusing different amounts of adhesive functional liquid into xerogels and kept inside a sealed bag. LIMBs were then stored at room temperature or transferred into a 4 °C fridge or a −80 °C freezer, depending on the testing storage condition. After a pre-determined storage period, LIMBs were taken out from the bag and tested for adhesion. In case of storage at −80 °C, LIMBs were let thaw at room temperature inside the bag for 5 min before use. No compression was used during sample preparation for peeling tests.

### Bacterial culture and viability assay

Gram-negative *Pseudomonas aeruginosa* (PAO1) and the Gram-positive bacteria *Staphylococcus aureus* (ATCC 25923) were used as model bacterial strains in this study. Fresh bacterial cultures were first prepared on nutrient agar from −80 °C glycerol stocks. Bacterial suspensions were then prepared by transferring single colonies from agar plates to Luria-Bertani broth and incubating the media at 37 °C and 200 rpm. Once the bacteria reached the exponential growth phase, they were harvested and centrifuged at 4000×*g* (Thermo Scientific, Heraeus Multifuge X3R). After discarding the supernatant, the cells were suspended in PBS. The optical density of bacteria at 600 nm (OD600) was set to 0.2 using a UV spectrophotometer (Thermo Scientific, Biomate 3S). The antimicrobial property was assessed by incubating the samples in 2 mL bacterial suspensions in a 12-well microtiter plate for 30 min. Pristine LIMB matrix and LIMB infused with 5% BZK solution in water were tested. The samples were gently rinsed with fresh PBS afterward to remove the loosely attached cells. The cells that remained attached to the samples were then assayed for viability by adding BacLight stains (Molecular Probes) prepared according to the manufacturer’s protocol. The BacLight kit contains SYTO 9, which produces fluorescent green color (excitation 483 nm/emission 503 nm) in cells with intact membranes (live cells), and propodeum iodide, which gives fluorescent red color (excitation 535/emission 617 nm) to cells with compromised membranes (dead/dying cells). After 15 min incubation in the presence of stains, the samples were observed under a confocal laser scanning microscope (Zeiss, LSM710) and at least 12 images were acquired for each substrate at various locations. The viability percentage of bacteria was calculated by dividing the number of live cells by the total number of cells.

### Burst pressure test

We manufactured a burst chamber following the ASTM F2392: Standard test method for burst strength of surgical sealants. Porcine heart tissue was trimmed into circles (30 mm in diameter, thickness of ca. 2 mm). A hole with 3 mm in diameter was then created in the center of the circular tissue sample using a biopsy punch. Xerogels were prepared into disk shapes (15 mm in diameter, 1.5 mm in thickness). For LIMBs with backings, a circular PET film (100 µm in thickness) was adhered to one side of the xerogel using super glue. The adhesive functional liquid was first infused into xerogel (25%-hydrated) to form LIMB. To initate the sealing, LIMB and defected tissues were brought into contact to completely cover defected area, without applying compression. The specimens were kept at 4 °C overnight inside a humidified environment before testing. During the test, a syringe was used to feed water to the burst chamber and to reach the defect. A pressure gauge was connected to the feeding tube to monitor the hydraulic pressure. The peak pressure that causes water leakage or burst through the defect was used as the burst pressure.

### In vivo biocompatibility and biodegradation

This experiment was approved by the McGill University Animal Care Committee (Protocol # 2019-8098) and performed according to the guidelines of the Canadian Council on Animal Care. The sample size of animal experiments is chosen on the basis of published literature on similar evaluations^8,22,33^. Female Sprague Dawley rats (250–300 g, Charles River Laboratories) were used for all the in vivo rat studies. Before implantation, both NBs and LIMBs (25%-hydrated with the adhesive functional liquid) were prepared into disk shape using aseptic techniques. The size was 5 mm in diameter and 2 mm in thickness. For implantation in the dorsal subcutaneous space, rats were anesthetized using isoflurane (4% isoflurane in oxygen) in an induction chamber. Anesthesia was maintained at 2% isoflurane using a nose cone during the surgery. A volume of 1 mL of saline was injected subcutaneously. Hair was removed, and the rats were placed over a heating pad during the surgery. The subcutaneous space was accessed by a 1-cm skin incision per implant in the rat’s back. Blunt dissection was performed from the incision point towards the shoulder blades of the rat to create subcutaneous space for implantation. NBs and LIMBs were implanted into the subcutaneous spaces. Up to four implants were placed per rat. The skin incisions were closed with sutures. On Day 3, 7, and 28, rats were euthanized by 5% isoflurane induction followed by CO_2_ inhalation. Subcutaneous regions of interest were excised and fixed in 4% paraformaldehyde solution for 48 h for histological analysis.

For in vivo biodegradation, FITC-labeled LIMBs were used for biodistribution imaging. During sample harvest for the biocompatibility experiment, major organs (liver, kidney, lung, heart, and spleen) of rats were collected at the same time and imaged using an IVIS spectrofluorophotometer to show the clearance pathway.

### Rat liver puncture bleeding model

This experiment was approved by the McGill University Animal Care Committee (Protocol # 2019-8098) and performed according to the guidelines of the Canadian Council on Animal Care. For hemostatic sealing of the volumetric hepatic injury, the rats were anesthetized using isoflurane (4% isoflurane in oxygen) in an induction chamber. Anesthesia was maintained at 2% isoflurane using a nose cone during the surgery. A volume of 1 mL of saline was injected subcutaneously. Abdominal hair was removed, and the rats were placed over a heating pad for the duration of the surgery. The liver was exposed via a laparotomy. A volumetric injury of 4 mm diameter and 3 mm depth was made to the liver using a biopsy punch. Cylindrical-shaped SURGIFOAM (Ethicon) or LIMB of 5 mm diameter and 4 mm depth was inserted into the wound immediately. The amount of blood loss until hemostasis was reached and the time to hemostasis was recorded for each group. After the hemostatic sealing was confirmed, the incision was closed using sutures. Two weeks after the implantation, the rats were euthanized by 5% isoflurane induction followed by CO_2_ inhalation. Livers with the implants were excised and fixed in 4% paraformaldehyde solution for 48 h for histological analysis.

### Rat liver incision bleeding model

This experiment was approved by the McGill University Animal Care Committee (Protocol # 2019-8098) and performed according to the guidelines of the Canadian Council on Animal Care. For hemostatic sealing of the deep incisional hepatic injury, the rats were anesthetized using isoflurane (4% isoflurane in oxygen) in an induction chamber. Anesthesia was maintained at 2% isoflurane using a nose cone during the surgery. A volume of 1 mL of saline was injected subcutaneously. Abdominal hair was removed, and the rats were placed over a heating pad for the duration of the surgery. The liver was exposed via a laparotomy. A laceration wound of 7 mm length and 3 mm depth was made to the liver using a #11 scalpel. Immediately after wiping off the blood using gauze, a pre-weighed LIMB, NB, or commercial hemostatic agent was applied to the site of the lesion. Hemostat was held in place by hand under some conditions without applying compression to the wound. The amount of blood loss until hemostasis was reached and the time to hemostasis was recorded for each group. The bleeding time of LIMB was only checked after 2 mins and not before. After the surgery, the rats were euthanized by 5% isoflurane induction followed by CO_2_ inhalation.

### Pig liver incision bleeding model

This experiment was approved by the University of British Columbia Animal Care Committee (Protocol #A18–0348) and performed according to the guidelines of the Canadian Council on Animal Care. All procedures were performed with the animals under general anesthesia. Female pigs (3-month-old, 40–50 kg) were induced with 4% isoflurane, endotracheally intubated, and mechanically ventilated at 10–12 breaths/min. Anesthesia was maintained with 1–3% isoflurane in combination with propofol (2–7 mg/kg/h) and midazolam (0.4–0.7 mg/kg/h). Long, central IV catheters were placed in both ears for drug and fluid administration. An intraarterial catheter was placed in both the hind leg pedal arteries for invasive blood pressure measurement, and these catheters were taped and bandaged in place to allow for blood collection throughout the recovery period. The temperature was maintained at 38.5–39.5 °C with a heating pad and was monitored using a rectal temperature probe. Hydration was maintained with 1.25% dextrose in isolyte solution administered intravenously 3–5 ml/kg/hr.

With the animal in dorsal recumbency, a laparotomy was performed. Liver lacerations that were 1.5 cm long and 1 cm deep were created using a scalpel. Plain gauze or LIMB was applied gently to the injury site without additional manual compression. Blood loss was quantified by placing pre-weighed gauze into the abdominal cavity and measuring the change in mass of the gauze. Time to hemostasis was measured. The monitoring period lasted 10 min, after which time manual compression was used to definitively stop the bleed so that additional injuries could be made to the pig. We tried to maximize the number of injuries on each pig up to three, or until the invasive blood pressure measurement from the hind leg pedal arteries dropped below the normal range, whichever occurred first.

### Histology and analysis

Fixed tissue samples were placed into 70% ethanol and submitted for histological processing and H&E staining at the Histology Core at McGill University. Z.-H.G. is the pathologist-in-chief at the University of British Columbia examined all histological sections. Representative images of each group were shown in the corresponding figures.

### Statistical analysis

A sample size of *N* ≥ 3 was used for all experiments. Data are shown as mean ± s.d. Statistical analysis was performed using one-way ANOVA and post hoc Tukey tests for multiple comparisons or Student’s *t*-tests for comparison between two groups (Prism 9). *P* values < 0.05 were considered statistically significant.

### Reporting summary

Further information on research design is available in the Nature Research Reporting Summary linked to this article.

## Supplementary information

Supplementary Information

Description of Additional Supplementary Files

Supplementary Movie 1

Supplementary Movie 2

Supplementary Movie 3

Supplementary Movie 4

Supplementary Movie 5

Supplementary Movie 6

Reporting Summary

## Data Availability

All data supporting the findings of this study are available within the Article and its Supplementary Information. Additional raw datasets generated during the study are too large to be publicly shared, yet they are available from the corresponding authors on reasonable request.
