# Supplementary material for: Liquid-infused microstructured bioadhesives halt non-compressible hemorrhage
Source: Nat Commun. 2022 Aug 26;13:5035. doi: 10.1038/s41467-022-32803-1 (PMC9418157; doi:10.1038/s41467-022-32803-1)
Supplement: Supplementary file 1 — Supplementary Information [file 41467_2022_32803_MOESM1_ESM.pdf]

Supplementary Information

Liquid-infused microstructured bioadhesives halt non-compressible hemorrhage

Guangyu Bao[1], Qiman Gao[1,2], Massimo Cau[3], Nabil Ali-Mohamad[3], Mitchell Strong[1], Shuaibing Jiang[1], Zhen Yang[1], Amin Valiei[4], Zhenwei Ma[1], Marco Amabili[1], Zu-Hua Gao[5], Luc Mongeau[1], Christian Kastrup[3,6,7,8,9,10] and Jianyu Li[1,11,12]**

Affiliations
[1]Department of Mechanical Engineering, McGill University, Montreal, Quebec, Canada
[2]Faculty of Dentistry, McGill University, Montreal, Quebec, Canada
[3]Michael Smith Laboratories, University of British Columbia, Vancouver, British Columbia, Canada
[4]Department of Chemical Engineering, McGill University, Montreal, Quebec, Canada
[5]Department of Pathology and Laboratory Medicine, University of British Columbia, Vancouver, British Columbia, Canada
[6]Blood Research Institute, Versiti, Milwaukee, Wisconsin, USA
[7]Department of Surgery, Division of Trauma and Acute Care Surgery, Medical College of Wisconsin, Milwaukee, Wisconsin, USA
[8]Department of Biochemistry, Medical College of Wisconsin, Milwaukee, Wisconsin, USA
[9]Department of Biomedical Engineering, Medical College of Wisconsin, Milwaukee, Wisconsin, USA
[10]Department of Pharmacology and Toxicology, Medical College of Wisconsin, Milwaukee, Wisconsin, USA
[11]Department of Biomedical Engineering, McGill University, Montreal, Quebec, Canada
[12]Department of Surgery, McGill University, Montreal, Quebec, Canada

Emails: jianyu.li@mcgill.ca (Jianyu Li); ckastrup@versiti.org (Christian Kastrup)

List of supplementary information:

Supplementary Fig. 1 | LIMB preparation.
Supplementary Fig. 2 | Porous structure of LIMB matrices.
Supplementary Fig. 3 | Pure shear test of 2M-LIMB.
Supplementary Fig. 4 | Cyclic tensile tests of LIMBs.
Supplementary Fig. 5 | Scaling between surface pore size and porosity of LIMB.
Supplementary Fig. 6 | Red blood cells are distributed through the cross-section of LIMB after absorption.
Supplementary Fig. 7 | Macroscopic investigation of the adhesion performance of LIMB and NB on dry and wet liver surfaces.
Supplementary Fig. 8 | Apply LIMB to a blood-exposed porcine heart without compression and then peel it off.
Supplementary Fig. 9 | Adhesion formation speed is tunable by the hydration state of LIMB.
Supplementary Fig. 10 | Effect of the viscosity of the adhesive functional liquid on bioadhesion performance.

Supplementary Fig. 11 | Compatibility between infiltration liquid and LIMB matrix affects the activity of reagents.
Supplementary Fig. 12 | Storage effect of LIMB at room temperature.
Supplementary Fig. 13 | Digital photos of LIMB before and after being immersed in PBS for 7 days.
Supplementary Fig. 14 | Macroscopic investigation of implanted NBs and LIMBs.
Supplementary Fig. 15 | Size change (in diameter) of FITC-LIMBs over 28 days of implantation.
Supplementary Fig. 16 | H&E staining of organs after implantation of LIMB for 28 days.
Supplementary Fig. 17 | Use of implantable hemostats to halt non-compressible hemorrhage.
Supplementary Fig. 18 | Digital photos showing the application of different hemostats to halt hemorrhage from rat liver incision.
Supplementary Fig. 19 | Use of LIMB to halt hemorrhage from a liver incision injury on pig.
Supplementary Fig. 20 | On-demand removal of LIMB after use.
Supplementary Fig. 21 | Adhesion energy of LIMB after being immersed in different removal agents.
Supplementary Table 1 | In vivo evaluation for the toxicity of EDC-based bioadhesives in literature.

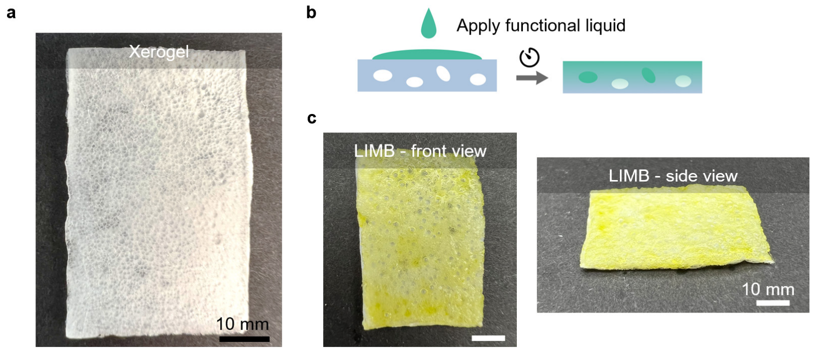

Supplementary Fig. 1 | LIMB preparation. a, Macroscopic images of xerogel and LIMB. **b**, Schematic showing the liquid infusion process. **c**, Front and side views of LIMB, with 25% of its volume filled with FITC-labeled adhesive functional liquid.

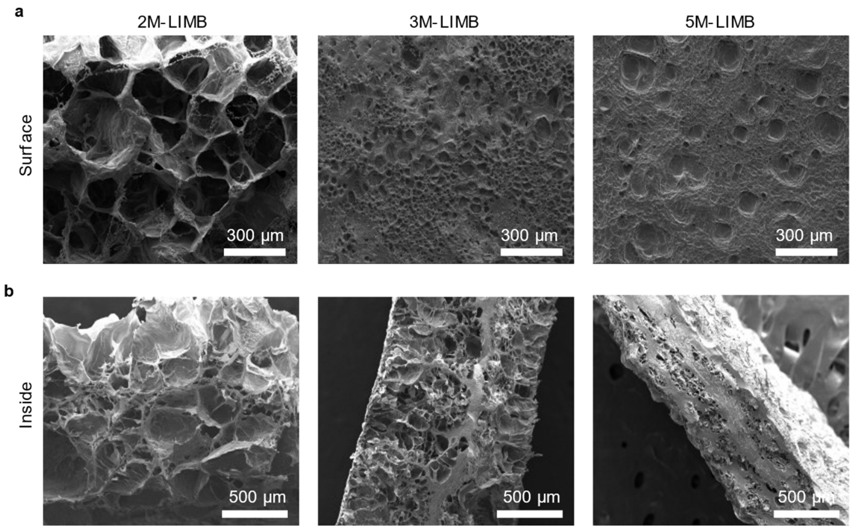

Supplementary Fig. 2 | Porous structure of LIMB matrices. a, Surface structure. **b**, Inside showing cross-sections. The experiment was repeated four times independently with similar results**.**

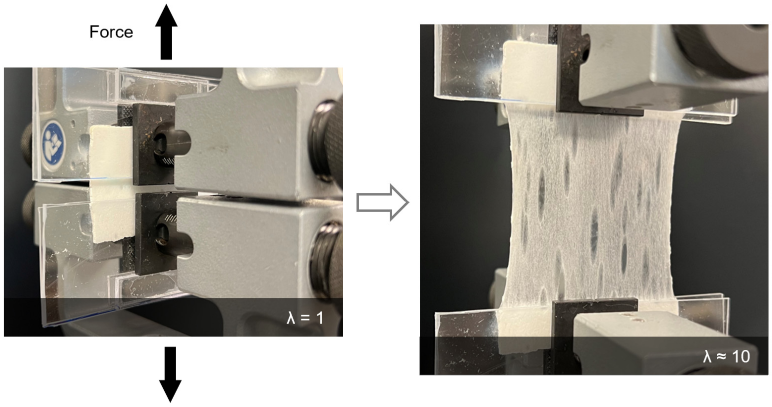

Supplementary Fig. 3 | Pure shear test of 2M-LIMB. The porous matrix is highly stretchable and insensitive to the pores.

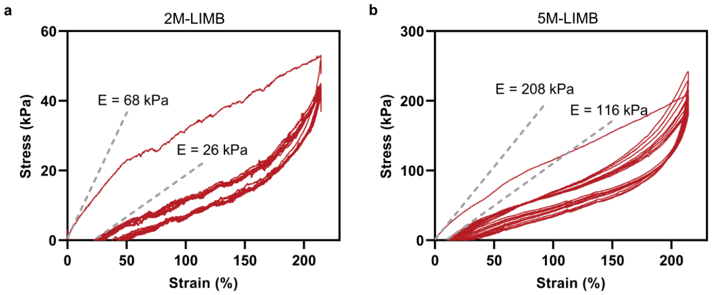

Supplementary Fig. 4 | Cyclic tensile tests of LIMBs. a, 2M-LIMB and **b**, 5M-LIMB at 25% hydration state.

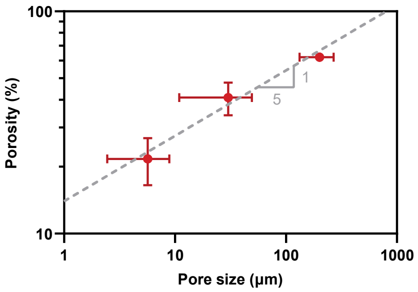

Supplementary Fig. 5 | Scaling between surface pore size and porosity of LIMB. Values represent the mean ± s.d. ($n = 3$).

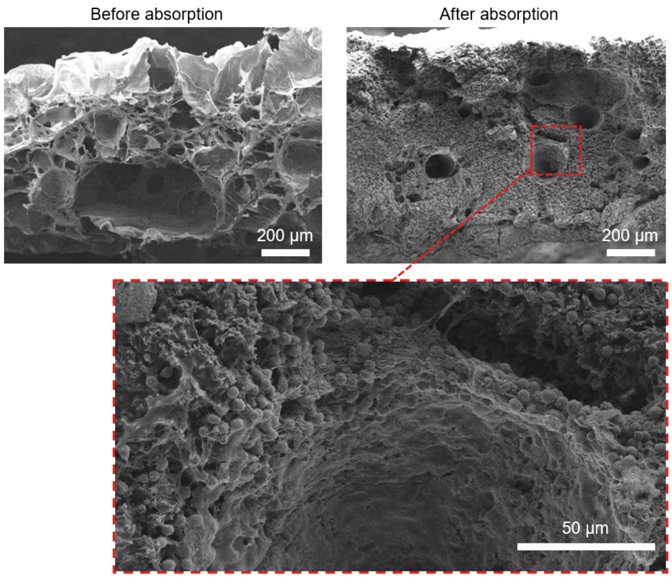

Supplementary Fig. 6 | Red blood cells are distributed through the cross-section of LIMB before and after absorption. The granular objects in the zoom-in image are red blood cells. The experiment was repeated four times independently with similar results.

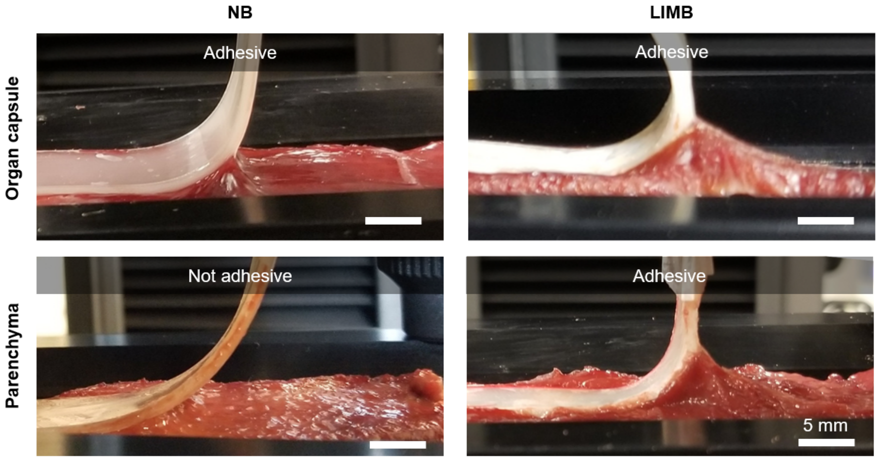

Supplementary Fig. 7 | Macroscopic investigation of the adhesion performance of LIMB and NB on dry and wet liver surfaces.

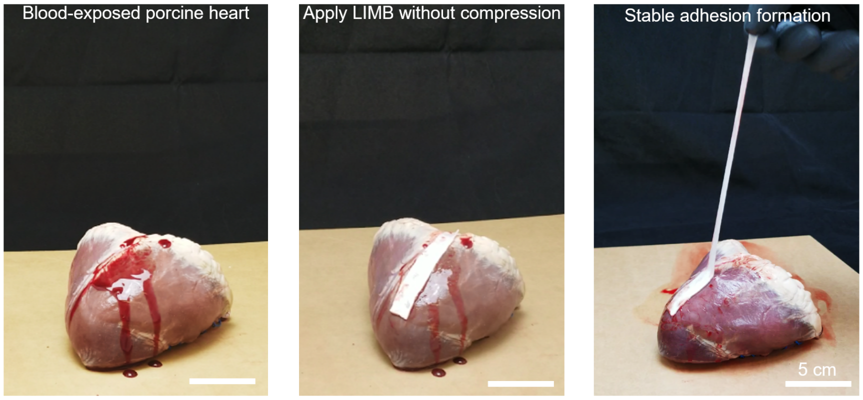

Supplementary Fig. 8 | Apply LIMB to a blood-exposed porcine heart without compression and then peel it off.

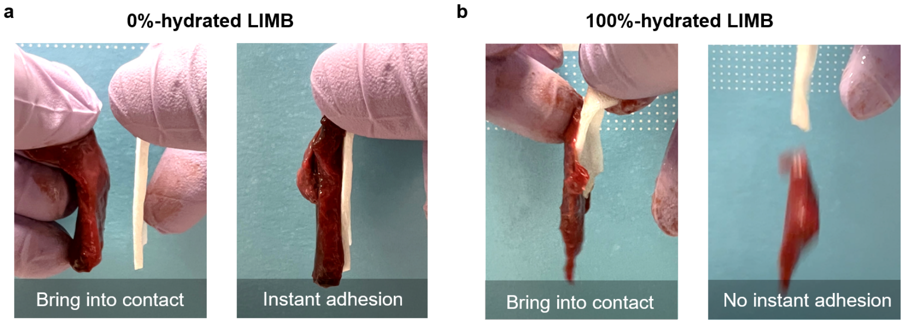

Supplementary Fig. 9 | Adhesion formation speed is tunable by the hydration state of LIMB. A wet liver parenchyma is brought into contact with LIMB of **a,** 0%-hydration and **b**, 100%-hydration and released immediately. 0%-hydrated LIMB forms instant adhesion with the tissue while no instant adhesion for 100%-hydrated LIMB. No compression is applied during the contact.

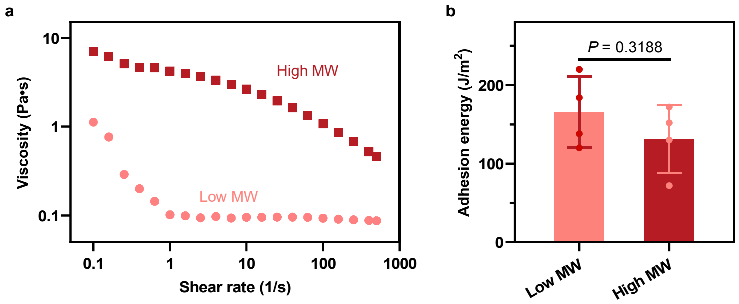

Supplementary Fig. 10 | Effect of the viscosity of the adhesive functional liquid on bioadhesion performance. a, Viscosity of adhesive functional liquids prepared by chitosan with low and high molecular weight (MW). **b**, Adhesion energy of LIMB on model tissues (collagen casing) when infused with adhesive functional liquid of different viscosities. No pressure was applied during the sample preparation. Values in **b** represent the mean ± s.d. (n = 4). Statistical significance and P values were determined by two-sided t-test.

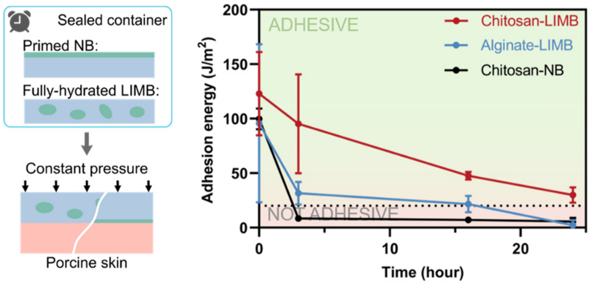

Supplementary Fig. 11 | Compatibility between infiltration liquid and LIMB matrix affects the activity of reagents. Adhesion energy above 20 J m^{-2} is determined as "adhesive" and below this value as "not adhesive". Values represent the mean ± s.d. (n = 4).

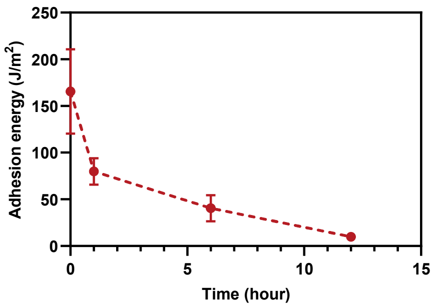

Supplementary Fig. 12 | Storage effect of LIMB at room temperature. The adhesion energy was measured by attaching 25% hydrated LIMB, after being stored at room temperature in a sealed container for pre-determined periods, on model tissue collagen casing. Values represent the mean ± s.d. (n = 4).

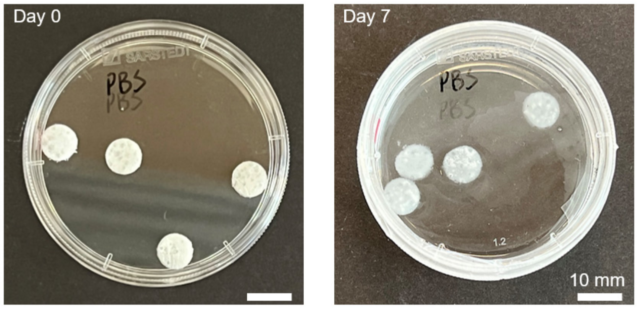

Supplementary Fig. 13 | Digital photos of LIMB before and after being immersed in PBS for 7 days.

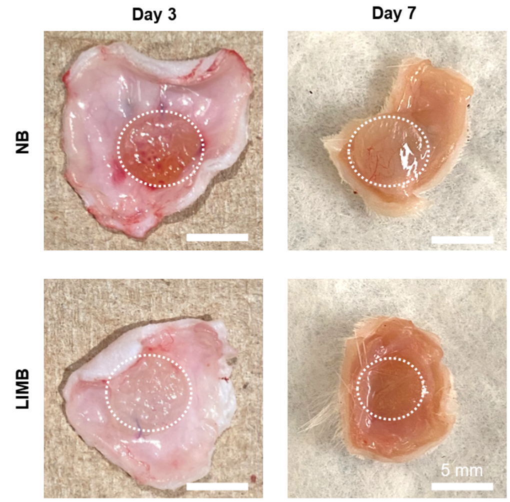

Supplementary Fig. 14 | Macroscopic investigation of implanted NBs and LIMBs. White circles indicate implants. LIMBs without FITC-label were used here.

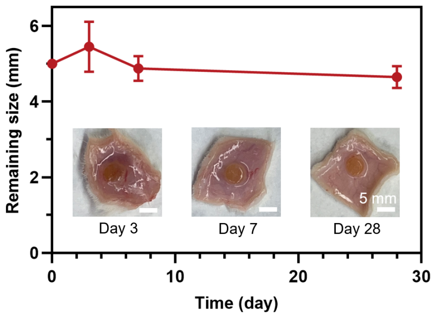

Supplementary Fig. 15 | Size change (in diameter) of FITC-LIMBs over 28 days of implantation. Values represent the mean ± s.d. (n = 4).

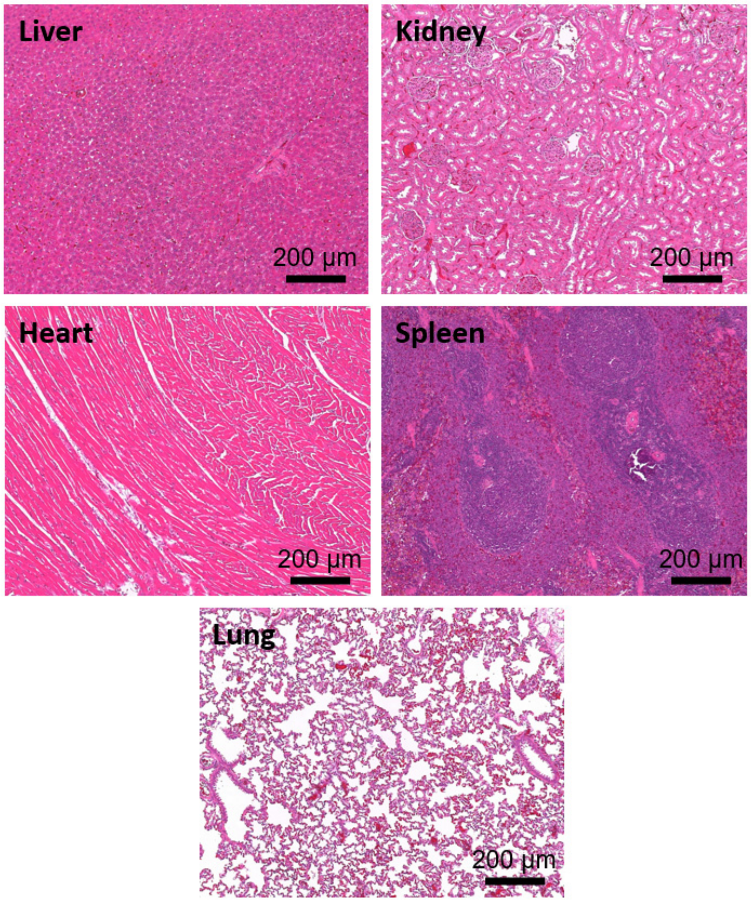

Supplementary Fig. 16 | H&E staining of organs after implantation of LIMB for 28 days. No toxicity was found from histological analysis. The experiment was repeated four times independently with similar results.

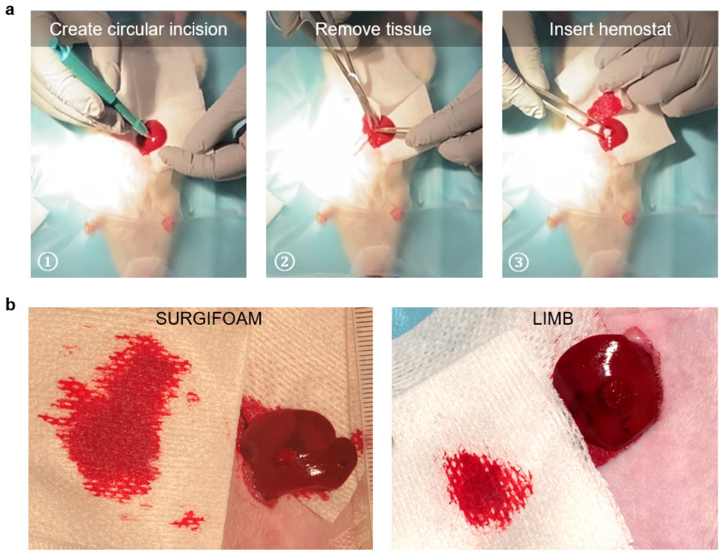

Supplementary Fig. 17 | Use of implantable hemostats to halt non-compressible hemorrhage. a, A 4-mm diameter, 3-mm deep volumetric liver injury was made in a rat using a biopsy punch and scissors. Hemostat was inserted into the bleeding site to halt the hemorrhage. **b,** Representative images showing the bleeding amount after applying commercially available SURGIFOAM and LIMB.

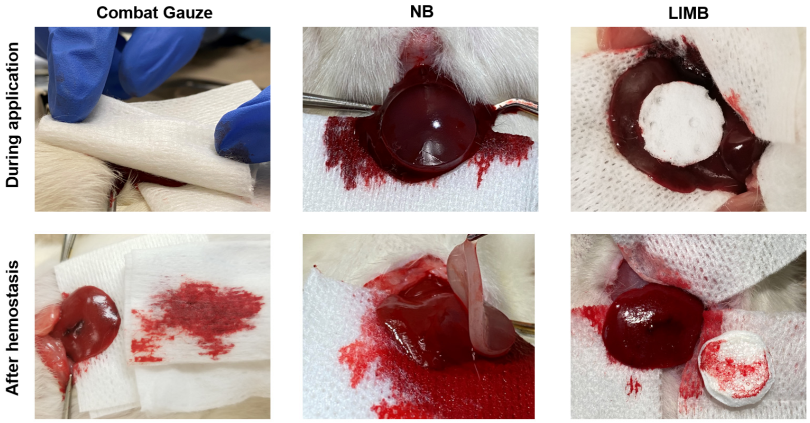

Supplementary Fig. 18 | Digital photos showing the application of different hemostats to halt hemorrhage from rat liver incision.

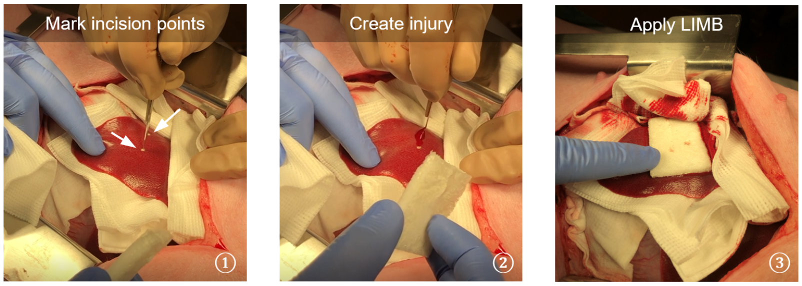

Supplementary Fig. 19 | Use of LIMB to halt hemorrhage from a liver incision injury on pig.

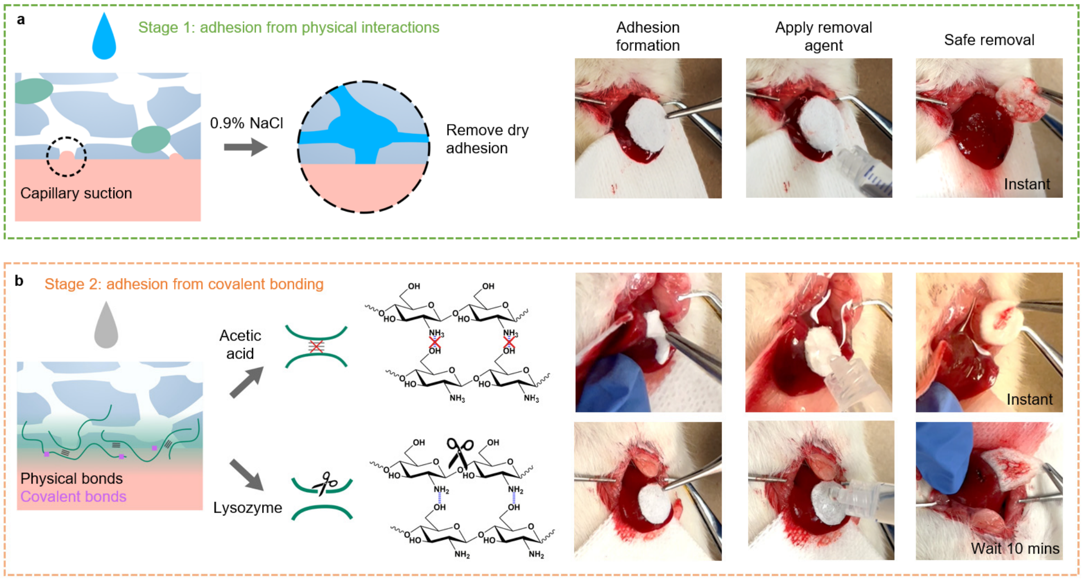

Supplementary Fig. 20 | On-demand removal of LIMB after use. a, Remove LIMB within 2-minute of placement on a bleeding rat liver. At this stage, the adhesion mainly comes from the capillary suction from the dry matrix. Using 0.9% NaCl solution to wet LIMB can achieve instant detachment. **b,** Remove LIMB after 10 mins of placement on a bleeding rat liver. Wetting alone at this stage cannot release LIMB as covalent bonds form. Using acetic acid solution (0.1 M) can disrupt the physical bonds from chitosan chains and instantly release LIMB from the tissue. Lysozyme solution (75 mg/mL) can also noticeably reduce the adhesion but needs 10 minutes to take effect.

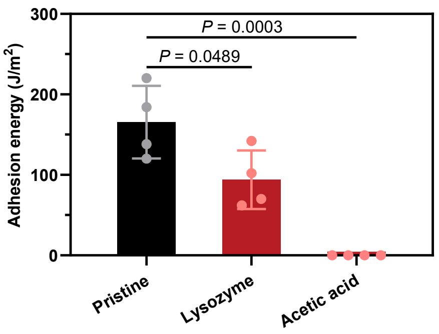

Supplementary Fig. 21 | Adhesion energy of LIMB after being immersed in different removal agents. LIMBs were first adhered to a model tissue (collagen casing) to form pristine samples. For lysozyme condition, pristine samples were immersed in a lysozyme solution (75 mg/mL in PBS) for 10 mins before peeling tests. For acetic acid conditions, pristine samples were immersed in a 0.1 M acetic acid solution for 5 seconds before peeling tests. Values represent the mean ± s.d. ($n = 4$). Statistical significance and P values were determined by two-sided Student t-test.

Supplementary Table 1 | In vivo evaluation for the toxicity of EDC-based bioadhesives in literature.

References	EDC concentration	Animal species	Implantation time	Implantation site	Toxicity
This work	20 mg/mL	Rat	2 weeks	Subcutaneous and liver	Very mild immune response
Science, **2017**. 357: 378-381	40 mg/mL	Rat	2 weeks	Subcutaneous, heart, and liver	Low cytotoxicity; Mild to moderate immune response; Lower toxicity compared to commercial products, such as SURGIFLOW (Ethicon), CoSeal (Baxter), and cyanoacrylate.
Advanced Materials, **2021**. 33: 2008553	12 mg/mL	Mouse	16 weeks	Subcutaneous and skin	Mild immune response; No systematic toxicity
Nature Biomedical Engineering, **2022**. 10.1038/s41551-021-00810-0	12 mg/mL	Rat	3 weeks	Tendon	No toxicity reported
Bioactive Materials, **2022**. 13: 260-268	2.4 mg/mL	Rat and pig	28 days (rat)<br>8 days (pig)	Skin (rat)<br>Liver (pig)	No toxicity reported
Science Advances, **2019**. 5: eaaw3963	12 mg/mL	Mouse	1 week	Skin	Mild to moderate immune response
