# Supplementary material for: Liquid-infused microstructured bioadhesives halt non-compressible hemorrhage
Source: Nat Commun. 2022 Aug 26;13:5035. doi: 10.1038/s41467-022-32803-1 (PMC9418157; doi:10.1038/s41467-022-32803-1)
Supplement: Supplementary file 2 — Description of Additional Supplementary Files [file 41467_2022_32803_MOESM2_ESM.pdf]

Description of Additional Supplementary Files

Supplementary Movie 1: Pressure-free adhesion of LIMB on a blood-exposed porcine heart.

Supplementary Movie 2: Peel off a LIMB from porcine heart tissue after immersing in PBS for 8 days.

Supplementary Movie 3: LIMB can be instantly released by 0.9% NaCl within 2 mins of placement.

Supplementary Movie 4: LIMB cannot be released by saline after covalent bond formation.

Supplementary Movie 5: Instant removal of LIMB using acetic acid after covalent bond formation.

Supplementary Movie 6: Removal of LIMB using lysozyme after covalent bond formation.
