# Supplementary material for: Liquid-infused microstructured bioadhesives halt non-compressible hemorrhage
Source: Nat Commun. 2022 Aug 26;13:5035. doi: 10.1038/s41467-022-32803-1 (PMC9418157; doi:10.1038/s41467-022-32803-1)
Supplement: Supplementary file 9 — Reporting Summary [file 41467_2022_32803_MOESM9_ESM.pdf]

nature portfolio

Corresponding author(s): Jianyu Li; Christian Kastrup

Last updated by author(s): Jul 28, 2022

Reporting Summary

Nature Portfolio wishes to improve the reproducibility of the work that we publish. This form provides structure for consistency and transparency in reporting. For further information on Nature Portfolio policies, see our Editorial Policies and the Editorial Policy Checklist.

Statistics

For all statistical analyses, confirm that the following items are present in the figure legend, table legend, main text, or Methods section.

n/a	Confirmed	
☐	☒	The exact sample size (*n*) for each experimental group/condition, given as a discrete number and unit of measurement
☐	☒	A statement on whether measurements were taken from distinct samples or whether the same sample was measured repeatedly
☐	☒	The statistical test(s) used AND whether they are one- or two-sided<br>*Only common tests should be described solely by name; describe more complex techniques in the Methods section.*
☒	☐	A description of all covariates tested
☐	☒	A description of any assumptions or corrections, such as tests of normality and adjustment for multiple comparisons
☐	☒	A full description of the statistical parameters including central tendency (e.g. means) or other basic estimates (e.g. regression coefficient) AND variation (e.g. standard deviation) or associated estimates of uncertainty (e.g. confidence intervals)
☐	☒	For null hypothesis testing, the test statistic (e.g. *F*, *t*, *r*) with confidence intervals, effect sizes, degrees of freedom and *P* value noted<br>*Give P values as exact values whenever suitable.*
☒	☐	For Bayesian analysis, information on the choice of priors and Markov chain Monte Carlo settings
☒	☐	For hierarchical and complex designs, identification of the appropriate level for tests and full reporting of outcomes
☒	☐	Estimates of effect sizes (e.g. Cohen's *d*, Pearson's *r*), indicating how they were calculated

Our web collection on statistics for biologists contains articles on many of the points above.

Software and code

Policy information about availability of computer code

Data collection	No software was used for data collection.
Data analysis	Microscopic images were analyzed by using ImageJ (1.53q). All statistical analyses were performed by using GraphPad Prism 9.

For manuscripts utilizing custom algorithms or software that are central to the research but not yet described in published literature, software must be made available to editors and reviewers. We strongly encourage code deposition in a community repository (e.g. GitHub). See the Nature Portfolio guidelines for submitting code & software for further information.

Data

Policy information about availability of data

All manuscripts must include a data availability statement. This statement should provide the following information, where applicable:

- Accession codes, unique identifiers, or web links for publicly available datasets
- A description of any restrictions on data availability
- For clinical datasets or third party data, please ensure that the statement adheres to our policy

All the data have been included in the paper and the Supplementary Information. Additional raw datasets generated during the study are too large to be publicly shared, yet they are available from the corresponding authors on reasonable request.

Field-specific reporting

Please select the one below that is the best fit for your research. If you are not sure, read the appropriate sections before making your selection.

☒ Life sciences ☐ Behavioural & social sciences ☐ Ecological, evolutionary & environmental sciences

For a reference copy of the document with all sections, see nature.com/documents/nr-reporting-summary-flat.pdf

Life sciences study design

All studies must disclose on these points even when the disclosure is negative.

Sample size	In vivo experiments on rats were conducted to investigate the in vivo biocompatibility and hemostatic efficiency by LIMB. A sample size of n = 4-6 was chosen on the basis of published literature of similar evaluations (Nature 575, 169–174 (2019)).<br><br>In vivo experiments on pigs were conducted to investigate the in vivo hemostatic efficiency by LIMB. A sample size of n = 6-7 was chosen on the basis of published literature of similar evaluations (Sci. Adv. 7, eabc9992 (2021)). We tried to maximize the number of injuries on each pig up to three, or until the invasive blood pressure measurement from the hind leg pedal arteries drops below the normal range, whichever came first.<br><br>Sample sizes of n ≥ 3 were used for all other experiments, following the convention. No sample size calculation was performed.
Data exclusions	No animal was excluded.
Replication	The in vivo biocompatibility study was reliably reproduced on the basis of a comparable histological assessment for each case by a blinded pathologist. The in vivo studies of hemostatic efficiency of the liver were reliably reproduced in different animals. All in vivo studies were independently performed at different universities with at least 1 day between surgeries. All in vitro studies were also independently performed with at least 1 day between experiments. All attempts at replication were successful.
Randomization	All the tests were performed with randomly allocated experimental groups.
Blinding	The histological assessments were conducted by a blinded pathologist on the basis of randomly mixed histological slides without information of type or study group. All measurements were conducted in a blinded fashion.

Reporting for specific materials, systems and methods

We require information from authors about some types of materials, experimental systems and methods used in many studies. Here, indicate whether each material, system or method listed is relevant to your study. If you are not sure if a list item applies to your research, read the appropriate section before selecting a response.

Materials & experimental systems

n/a	Involved in the study	
☒	☐	Antibodies
☐	☒	Eukaryotic cell lines
☒	☐	Palaeontology and archaeology
☐	☒	Animals and other organisms
☒	☐	Human research participants
☒	☐	Clinical data
☒	☐	Dual use research of concern

Methods

n/a	Involved in the study	
☒	☐	ChIP-seq
☒	☐	Flow cytometry
☒	☐	MRI-based neuroimaging

Eukaryotic cell lines

Policy information about cell lines

Cell line source(s)	Human vocal fold fibroblasts, provided by Dr. Susan Thibeault from University of Wisconsin-Madison.
Authentication	The cell line was authenticated by Dr. Susan Thibeault's team, on the basis of standard techniques including morphology check, cytogenetic analysis, and mycoplasma detection (Tissue Eng. Part C Methods 5, 201-212 (2009)).
Mycoplasma contamination	The cell line has been tested negative for mycoplasma contamination.
Commonly misidentified lines (See ICLAC register)	No commonly misidentified cell lines were used.

Animals and other organisms

Policy information about studies involving animals; ARRIVE guidelines recommended for reporting animal research

Laboratory animals	Rats (female, Sprague Dawley, 8-10 weeks, 250-300 g) and domestic pigs (female, 3 months, 40-50 kg).
Wild animals	The study did not involve wild animals.
Field-collected samples	The study did not involve samples collected from the field.
Ethics oversight	The rat protocol was approved by the McGill University Animal Care Committee (Protocol # 2019-8098). The pig protocol was approved by the University of British Columbia Animal Care Committee (Protocol #A18–0348). All the procedures were performed according to the guidelines of the Canadian Council on Animal Care.

Note that full information on the approval of the study protocol must also be provided in the manuscript.
